# Design of symmetric TIM barrel proteins from first principles

**DOI:** 10.1186/s12858-015-0047-4

**Published:** 2015-08-12

**Authors:** Deepesh Nagarajan, Geeta Deka, Megha Rao

**Affiliations:** Biochemistry Department, Indian Institute of Science, Bangalore, India; Molecular Biology Unit, Indian Institute of Science, Bangalore, India

## Abstract

**Background:**

Computational protein design is a rapidly maturing field within structural biology, with the goal of designing proteins with custom structures and functions. Such proteins could find widespread medical and industrial applications. Here, we have adapted algorithms from the Rosetta software suite to design much larger proteins, based on ideal geometric and topological criteria. Furthermore, we have developed techniques to incorporate symmetry into designed structures. For our first design attempt, we targeted the (α/β)_8_ TIM barrel scaffold. We gained novel insights into TIM barrel folding mechanisms from studying natural TIM barrel structures, and from analyzing previous TIM barrel design attempts.

**Methods:**

Computational protein design and analysis was performed using the Rosetta software suite and custom scripts. Genes encoding all designed proteins were synthesized and cloned on the pET20-b vector. Standard circular dichroism and gel chromatographic experiments were performed to determine protein biophysical characteristics. 1D NMR and 2D HSQC experiments were performed to determine protein structural characteristics.

**Results:**

Extensive protein design simulations coupled with ab initio modeling yielded several all-atom models of ideal, 4-fold symmetric TIM barrels. Four such models were experimentally characterized. The best designed structure (Symmetrin-1) contained a polar, histidine-rich pore, forming an extensive hydrogen bonding network. Symmetrin-1 was easily expressed and readily soluble. It showed circular dichroism spectra characteristic of well-folded alpha/beta proteins. Temperature melting experiments revealed cooperative and reversible unfolding, with a T_m_ of 44 °C and a Gibbs free energy of unfolding *(ΔG°)* of 8.0 kJ/mol. Urea denaturing experiments confirmed these observations, revealing a C_m_ of 1.6 M and a *ΔG°* of 8.3 kJ/mol. Symmetrin-1 adopted a monomeric conformation, with an apparent molecular weight of 32.12 kDa, and displayed well resolved 1D-NMR spectra. However, the HSQC spectrum revealed somewhat molten characteristics.

**Conclusions:**

Despite the detection of molten characteristics, the creation of a soluble, cooperatively folding protein represents an advancement over previous attempts at TIM barrel design. Strategies to further improve Symmetrin-1 are elaborated. Our techniques may be used to create other large, internally symmetric proteins.

**Electronic supplementary material:**

The online version of this article (doi:10.1186/s12858-015-0047-4) contains supplementary material, which is available to authorized users.

## Background

Finding a protein sequence that adopts a desired tertiary fold is the aim of de novo protein design. Besides increasing our understanding of protein energetics and folding mechanisms, de novo protein design also has the potential to create useful proteins with novel applications. Initial breakthroughs in the field involved the synthesis of stable peptides [1, 2] and stable 4-helix bundles [3–6]. Further advancements included the large-scale redesign of existing proteins [7], culminating in the creation of a protein that adopts a novel fold (Top7) [8]. Recent advances have included the large-scale de novo design of small alpha/beta proteins, based on computationally derived ideal topological principles [9]. While substantial progress has been made in designing small proteins of approximately 100 residues in length, the design of larger proteins, including those that adopt the TIM barrel fold, remains an open problem.

The TIM barrel is the most abundant tertiary fold, observed in 10 % of all known protein structures [10, 11]. This fold is believed to have originated through gene duplication and domain fusion, with a large majority of TIM barrels originating from such a common ancestor. The domain fusion hypothesis was first proposed for the imidazole glycerol phosphate synthase (HisF) and ProFAR isomerase (HisA) enzymes, based on internal twofold sequence and structural symmetry [12]. Consequently, the HisA-HisF system became the subject of intense protein engineering efforts, including the successful replication of domain fusion events. Examples include proteins (HisF-C*C and HisF-C***C) designed from C-terminal HisF half-barrels [13, 14], and the successful design of HisA-HisF chimeric TIM barrels [15]. Such constructs have even been restored to wild-type catalytic activity [16]. Other chimeric TIM barrels were designed from different folds, including HisF and the (βα)_5_-flavodoxin-like fold (CheY) [17, 18]. Such work normally involves extensive random mutagenesis and selection in order to create stable proteins. More recently, however, a perfectly symmetric TIM barrel protein (FLR) was computationally designed from HisF fragments using the Rosetta software suite [19].

Despite its evolutionary success and despite successful chimeric designs from pre-existing proteins, attempts to design TIM barrels from scratch have met with mixed results. Early attempts at sequence-based TIM barrel design included the proteins Octarellin I,II and III [20–22]. Later structure-based methods using all-atom models resulted in the Octarellin V and VI designs [23, 24]. While these proteins adopted an alpha/beta structure and showed some cooperative folding characteristics, they were insoluble and prone to aggregation. Experiments had to be performed on protein resolubilized from inclusion bodies.

In this work, we describe novel insights into TIM barrel folding mechanisms and their successful application to TIM barrel design. These insights were derived from studying natural TIM barrel structures and from previous design attempts. We describe techniques for the de novo design of large, symmetric proteins adopting ideal topologies, using the Rosetta software suite. By incorporating symmetry, we greatly reduce the sequence and conformational space searched, and hence computational resources required to design large proteins. As a test of our techniques, we created models for 224-residue long, 4-fold symmetric, geometrically and topologically ideal TIM barrels. We have experimentally characterized the *Symmetrin* family of proteins, and present circular dichroism, thermal denaturation, chemical denaturation, size-exclusion chromatography, and 1D NMR data. We show that our best designed TIM barrel protein (Symmetrin-1) was easily expressed, readily soluble, exhibited cooperative and reversible unfolding characteristics, adopted a monomeric conformation, and displayed a well resolved 1D NMR spectrum.

## Methods

### Creation of a representative subset of natural TIM barrels

In order to derive insights into TIM barrel stability and folding, we created a representative subset of all naturally occurring TIM barrel structures. All TIM barrel structures from the CATH database [25] (class: 3, architecture: 20) were downloaded. Structures containing multiple domains were removed. A non-redundant dataset (40 % sequence identity threshold) of all TIM barrel structures was created using the program CD-hit (version4.5.4) [26]. Visual inspection of this dataset was carried out to eliminate structures poorly conforming to the TIM barrel fold, including incompletely closed beta-barrels. All structures were subjected to one round of all-atom relaxation using the Rosetta software suite (version 3.5). The resulting dataset contained 215 proteins. A list of all chosen structures is provided in the supporting information (Additional file 1: Text S1). A semi-automatic, graph-theoretic classification algorithm was used to identify beta-barrels within this dataset, and classify residues as belonging to the core or pore. Pore residues point towards the beta-barrel origin and away from the alpha-beta interface, whereas core residues point away from the beta-barrel origin and towards the alpha-beta interface. Scripts for the classification algorithm are provided in the supporting information (Additional file 2: Protocol S1). The TIM barrel dataset and classification scheme were used for further statistical analyses.

### TIM barrel Topological design

**Fig. 1:**
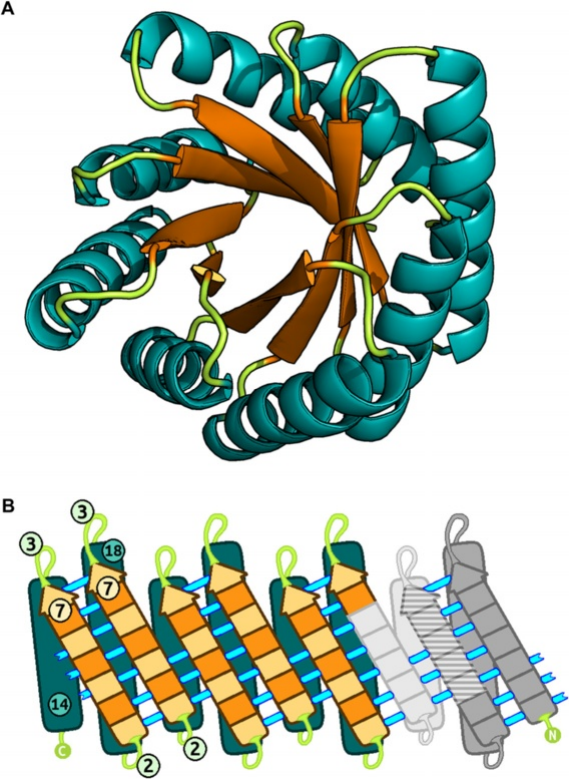
Depiction of the Symmetrin backbone and topology. **a** The backbone model used for all Symmetrin designs. Alpha helices are colored teal. Beta sheets are colored orange. Loops are colored green. The TIM barrel fold and designed four-fold symmetry are readily apparent. **b** Designed secondary structure topology. Helices are colored teal. Sheets are colored in two shades of orange. Lighter shades indicate residues pointing out of the page, towards the reader. Darker shades indicate residues pointing into the page, away from the reader. Cyan lines depict the beta-barrel's hydrogen bonding network. Numbers depict residues forming their respective secondary structures. The two ideal βαβ motifs chosen for this design are colored in two shades of grey. Residues shared by both βαβ motifs are colored in horizontal stripes. For clarity, only 2 of the 8 ideal βαβ motifs constituting the TIM barrel topology are colored

The canonical TIM barrel scaffold contains eight peripheral alpha helices, enclosing a parallel, eight-stranded beta-barrel. In such a scaffold, connecting loops would serve no catalytic functions, and their length is therefore restricted. Koga et. al. [9] described techniques to design proteins of diverse topologies from a small set of topologically ideal motifs. For example, a TIM barrel containing a repeating βαβα motif can be designed by selecting and integrating ideal βαβ motifs of appropriate sizes. Two ideal βαβ motifs [β(7)-l(3)-α(18)-l(2)-β(7) and β(5)-l(3)-α(14)-l(2)-β(5)] of differing sizes were integrated to form a repeating 56-residue βαβα motif [β(7)-l(3)-α(18)-l(2)-β(7)-l(3)-α(14)-l(2)]. Two βαβ motifs of different sizes were required in order to introduce a shear number of 8 [27]. The designed TIM barrel topology consisted of four βαβα repeats, resulting in a structure composed of 224 residues in total (Fig. 1b). A four-fold, rather than eight-fold, symmetry was adopted based on its compatibility with our chosen first principles, and based on previous attempts to design TIM barrels adopting such symmetries [20]. This topological design was used as the basis for the creation of a full-atom backbone model.

### Design of a geometrically ideal beta-barrel backbone

Ideal geometrical criteria [27] were used to design the beta-barrel backbone, excluding loops and alpha-helices. This backbone structure modeled Cα atoms, intrastrand and interstrand distances (3.3 Å and 4.4 Å, respectively). Defining intrastrand and interstrand distances requires a beta-vector, which is a line of best fit for the backbone atoms (N, Cα, C). The intrastrand distance is the distance between the coordinates of successive Cα atoms on the same beta strand projected onto the beta-vector. The interstrand distance is the shortest distance between two beta-vectors of neighboring, hydrogen-bonded beta strands (Additional file 3: Figure S1). The beta-barrel's characteristic tilt (37°), and the curvature along the axis of the beta-barrel at a defined radius (7.2 Å). Barrel ellipticity will not occur in a 4-fold symmetric TIM barrel, and therefore this term was ignored. In order to simplify this model, beta-sheet coiling was ignored. However, beta-sheet coiling was implicitly reintroduced later in the design protocol through the use of βαβ fragments derived from natural TIM barrel structures, and through Rosetta backbone energy-minimization protocols. A Cα-based model of the beta-barrel backbone is provided in the supporting information (Additional file 4: Text S2).

### Alpha/beta fragment based TIM barrel assembly

A full-atom library of βαβ fragments conforming to ideal topological criteria was derived from the CATH TIM barrel representative subset. Cα atoms of beta-sheets within these fragments were superposed onto the designed Cα-based beta-barrel backbone using Kabsch's algorithm [28]. Fragment rejoining and loop design were performed using cyclic coordinate descent application [29] included in Rosetta. All atomic distances were normalized using the idealization application included in Rosetta. Foldit [30] was used to visually inspect the structure, and to manually resolve clashes by altering rotamers and residue type. Coarse-grained backbone relaxation was performed using the all-atom *relax* application [31, 32] included in Rosetta, producing TIM barrel backbones ready for symmetric sequence design. 100 such TIM barrel backbones were constructed. The backbone with the best Ramachandaran angles which also maintained the designed secondary structure was selected as the starting scaffold for sequence design (Fig. 1a).

### Symmetric sequence design and all-atom relaxation

The protein design workflow used is similar to that described in previous work [9], but with considerable modifications. Two complementary applications included in Rosetta help design a sequence onto a starting scaffold. The *fixed backbone design* application [7, 8, 33–36] involves optimization of sidechain-rotamer placement upon a stationary backbone, creating a new amino-acid sequence to fit the original backbone. The *relax* application [31, 32] performs an all-atom structural refinement on a previously designed structure, perturbing both backbone and sidechain atoms. The alternate execution of fixed backbone design and relax applications on a starting scaffold is required to design a final protein sequence. Since both applications invoke stochastic simulated annealing algorithms, multiple sequences must be designed for any given backbone in order to reach the energy minima.

Residue assignment for the ideal scaffold was guided by both visual inspection using Foldit and by the use of Naccess (version2.1.1) [37]. All surface residues were assigned as hydrophilic. Residues with accessible surface areas of less than 30 % of the total were classified as buried. Buried residues at the alpha-alpha and alpha-beta interfaces were assigned as hydrophobic. Pore residues were assigned as both, but with bias for hydrophilic residues. Only loops were allowed to contain glycine. Cysteine residues were disallowed to prevent unwanted disulfide bond formation. Residue assignments were introduced into the fixed backbone design application using the *resfile* input format. A bias against hydrophobic residues was introduced by probabilistically altering the resfile input such that hydrophobic residues were requested at a given pore residue position with a probability of 0.26. Individual residue selection was performed autonomously by Rosetta applications.

We created a workflow that enforces symmetry during every round of design. The 224-residue starting scaffold was divided into four, 56-residue quadrants. After each round of fixed backbone design execution, a quadrant was randomly selected and its sequence transferred to all other quadrants. This symmetric structure was then passed through the relax application for further refinement. 16 such iterations were performed to design every structure, and 1000 such structures were designed. All designs were visually inspected using Foldit. Exposed hydrophobic residues and buried, non-hydrogen-bonded polar residues were mutated to their optimal identities using Foldit. Furthermore, the inbuilt *RosettaHoles* application [38] was used to eliminate structures possessing large internal voids. 40 structures possessing the lowest Rosetta scores were selected for ab initio modeling purposes.

### Ab initio modeling and the generation of folding landscapes

The fixed backbone design and relax applications of Rosetta are capable of designing protein structures close to a local energy minima (positive design). However, these applications are incapable of exploring the conformational landscape, and are incapable of detecting energetically favorable alternate conformations possibly closer to the global energy minima (negative design). The *ab initio modeling* application [39–43] included in Rosetta is therefore used for such purposes. The ab initio modeling application attempts to fold a protein based only on its sequence. Due to the stochastic nature of the simulated annealing algorithm, multiple models are generated for any given sequence. These models can be compared to the designed structure. A strongly funneled folding landscape is observed when a large fraction of well-scoring ab initio models have low Cα root mean squared deviations (RMSDs) when compared to the template structure.

**Fig. 2:**
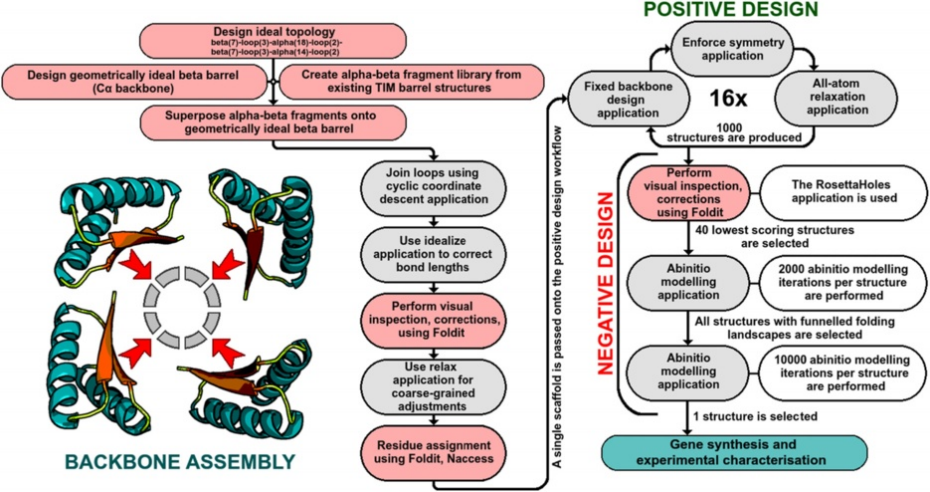
Protein design protocol used for symmetric protein design. All computational steps required to design a protein sequence ready for experimental characterization are depicted. Pink boxes indicate steps involving manual intervention. Grey boxes indicate completely automated steps

However, ab initio modeling is computationally intensive, scaling exponentially with increases in sequence length. Given current computational limitations, the application of ab initio protein folding is restricted to sequences of approximately 100 residues in length. We circumvented this limitation by modeling 95-residue (αβ)_3_ foldon-like modules for all selected structures. These modules contain all unique interactions present within the entire symmetric structure, and were therefore excellent proxies for the whole TIM barrel. 2000 ab initio folding simulations were performed on each of the 40 structures possessing the lowest Rosetta scores. Three structures displayed strongly funneled folding landscapes, and were each subjected to 10000 ab initio folding simulations. Of these three structures, one structure (Symmetrin-1) and three variants (Symmetrin-2,3,4) were selected for experimental characterization. The entire protein design protocol has been illustrated in Fig. 2. Scripts for the positive design workflow are provided in the supporting information (Additional file 5: Protocol S2).

### Protein expression and purification

Genes encoding the Symmetrin proteins were purchased from GeneWiz, Inc. The genes were synthesized along with a C-terminal GS-(H)x6 tag and cloned into the expression vector pET-20b, between the 5' NdeI and 3' XhoI restriction sites. The genes were then cloned and expressed in *E. coli* BL21 cells. Protein expression was performed using two techniques. For expressing non-labelled protein, transformed, isolated colonies of BL21 cells were inoculated into 50 ml LB media containing 100 mg/ml ampicillin and incubated at 37 °C/18 h, till an OD(600) of 1.0. This starter culture was inoculated into 1 l LB media containing 100 mg/ml ampicillin and 0.5 mM of isopropyl b-D-1-thiogalactopyranoside (IPTG), and incubated at 37 °C/8 h, till an OD(600) of 0.8. For ^15^ N labeled protein required for HSQC experiments, transformed, isolated colonies of BL21 cells were inoculated into 20 ml LB media containing 100 mg/ml ampicillin and incubated at 37 °C/18 h, till an OD(600) of 1.0. This starter culture was inoculated into 1 l M9 media containing ^15^NH_4_Cl as the sole nitrogen source, and 100 mg/ml ampicillin. This culture was incubated at 37 °C/4 h, till an OD(600) of 0.5. Induction was performed using 0.5 mM of isopropyl b-D-1-thiogalactopyranoside (IPTG), and incubated at 25 °C/4 h, till an OD(600) of 0.8.

For both techniques, the cells were harvested by centrifugation at 6000 rpm/10 min. Cells were resuspended in 30 ml of phosphate buffer (50 mM NaCl + 50 mM KH_2_PO_4_/K_2_HPO_4_ buffer, pH8.0). Subsequent purification steps were performed in this buffer.

Protein purification was performed using a Nickel-NTA resin (Ni-NTA Agarose, Nucleo-pore). 0.1 mg lysozyme and 10 μl of 100 mM phenylmethanesulfonylfluoride (PMSF) were added to the *E. coli* resuspension prior to sonication. Centrifugation at 10,000 rpm/30 min at 4 °C was performed, after which the supernatant was incubated with 0.5 ml Nickel-NTA resin (bed volume) for 1 h at 4 °C on a gel-rocker. A 10 ml Pierce centrifuge column (Thermo) was used to isolate the Nickel-NTA resin from the supernatant via gravity flow. The resin was washed with 20 ml phosphate buffer + 75 mM imidazole and eluted in six fractions of 0.5 ml phosphate buffer + 500 mM imidazole each. The fractions were analyzed on a 15 % SDS-PAGE gel, and fractions containing the protein of interest were repooled for further purification steps. Protein expression and solubility varied. Good Symmetrin-1, 2, and 3 expression was observed using the Bradford test (Fluka), at 10 mg/l. These proteins were found to be in the soluble fraction, and no subsequent solubilization steps were required. They were stable when stored at 4 °C at concentrations of 2.5 mg/ml. In contrast, Symmetrin-4 expressed poorly (3 mg/l) and was found in the insoluble fraction.

Soluble proteins were dialyzed in phosphate buffer (50 mM NaCl + 50 mM KH_2_PO_4_/K_2_HPO_4_ buffer, pH8.0) and reconcentrated using a Vivaspin centrifugal concentrator (10 kDa MWCO, GE healthcare) before performing further experiments.

### Circular dichroism experiments

All circular dichroism (CD) experiments were performed on the Jasco J-810 spectrophotometer. Protein concentrations used ranged from 10-50 μM. CD spectra were acquired within the far UV region (200-250 nm), and near UV region (250-320 nm) at 25 °C, and at a scan speed of 100 nm/min. Temperature melting curves were acquired at 222 nm, at temperature ranges between 10-95 °C, and at a scan speed of 1 °C/min. For far UV CD experiments, quartz cuvettes with a path length on 0.1 cm were used, whereas for near UV CD experiments, quartz cuvettes with a path length of 1 cm were used. Urea melting curves were acquired at 222 nm at 25 °C. They were titrated manually, and collected over a concentration gradient of 0 M-4 M urea at intervals of 0.25 M urea. All spectra were collected at a 3 nm bandwidth and a 4 s response time. All spectra were collected thrice, averaged, and corrected for buffer spectrum.

### Gel filtration experiments

Samples were concentrated to 2 ml using a Vivaspin centrifugal concentrator (10 kDa MWCO, GE healthcare), and dialyzed in a high-salt phosphate buffer (150 mM NaCl + 50 mM KH_2_PO_4_/K_2_HPO_4_ buffer, pH8.0) using SnakeSkin dialysis tubing (10 kDa MWCO, Thermo) prior to Size exclusion chromatography (SEC). A higher salt concentration was used in order to prevent any interaction of the proteins with the chromatography column's resin. SEC was performed using a 120 ml Superdex S75 FPLC column (GE healthcare). Proteins were dialyzed in phosphate buffer (50 mM NaCl + 50 mM KH_2_PO_4_/K_2_HPO_4_ buffer, pH8.0) and reconcentrated using a Vivaspin centrifugal concentrator (10 kDa MWCO, GE healthcare) for further experiments. The molecular markers used to calibrate S75 FPLC column were aprotinin (6.5 kDa), cytochrome c (12.4 kDa), carbonic anhydrase (29 kDa), albumin (66 kDa), and alcohol dehydrogenase (150 kDa).

### NMR experiments

All proteins were dialyzed against a low-salt phosphate buffer (50 mM NaCl + 20 mM KH_2_PO_4_/K_2_HPO_4_ buffer, pH8.0) before collecting their NMR spectra. 50 μl of deuterium oxide was added to protein samples at 500 μl/0.1 mM before collecting the NMR spectrum. NMR spectra were collected on the Bruker Avance III 700 Mhz high resolution, multinuclear FT-NMR spectrometer running under TOPSPIN 2.4 (ICON NMR 4.2 for automation).

### Thermodynamic calculations

Estimation of the Gibbs free energy of unfolding *(ΔG°)* was performed using standard methods, as described below. Experimentally, denaturation curves were obtained by measuring circular dichroism ellipticity (mdeg) against temperature or urea concentration gradients. Absolute ellipticity values were converted into fractional denaturation *(f*_*D*_*)* values using equation-1, where *S* is the ellipticity at a given temperature or denaturant concentration, *S*_*N*_ is the ellipticity of the native state (maximum ellipticity) , and *S*_*D*_ is the ellipticity of the denatured state (minimum ellipticity). The equilibrium constant at any given temperature or denaturant concentration was calculated using equation-2.

1 $$ {f}_D=\left(S-{S}_N\right)/{S}_D-{S}_N $$

2 $$ {K}_{eq}={f}_D/\left(1-{f}_D\right) $$

For thermal denaturation experiments, the transition region of the denaturation curve was used to construct a Van’t Hoff plot *[ln(K*_*eq*_*) vs. 1/T ]*. The Gibbs free energy of unfolding was calculated using equation-3, where *ΔH* represents enthalpy change, *ΔS* represents entropy change, *T* is the absolute temperature, *R* is the gas constant, *m* is the slope and *C* is the Y-intercept of the Van’t Hoff plot. Further, the melting temperature *(T*_*m*_*)* was calculated using equation-4.

3 $$ \varDelta G{}^{\circ}=\varDelta H-T\varDelta S=R\left(m+TC\right) $$

4 $$ {T}_m=\varDelta H/\varDelta S $$

For urea denaturation experiments, the energy of unfolding (*ΔG)* at any denaturant concentration is calculated using equation-5, where *R* is the gas constant and *T* is the absolute temperature. The transition region of the denaturation curve was used to construct a *[ΔG vs. urea conc (M)]* plot. The Gibbs free energy of unfolding was calculated using equation-6, where *C* represents the Y-intercept. Further, the melting concentration *(C*_*m*_*)* was calculated using equation-7, where *m* represents the slope.

5 $$ \varDelta G=-RT1\mathrm{n}\left({K}_{eq}\right)=-RT1\mathrm{n}\left({f}_D/\left(1-{f}_D\right)\right) $$

6 $$ \varDelta G{}^{\circ}=C $$

7 $$ {C}_m=-C/m $$

## Results and discussion

### Insights derived from natural TIM barrel structures

The TIM barrel scaffold contains two distinct buried regions, historically but inaccurately referred to as the *core* and *pore*. The core consists of all residues along the alpha-beta interface, exterior to the central beta-barrel. These residues are usually buried, with low solvent accessibility, and are mostly hydrophobic [44]. The pore consists of all interior beta-barrel residues, enclosed and surrounded by the beta-barrel backbone. These residues, like their core counterparts, are mostly buried. However, extensive hydrogen bonding and salt bridge networks have been reported to exist within the pore. In the case of the enolase barrel, a hydrogen bonding network traverses the entire length of the pore connecting the C-terminal active site with the N-terminal base [45]. Such networks were shown to remain conserved within protein families, and could even be used to infer evolutionary relationships between TIM barrel structures [46].

**Fig. 3:**
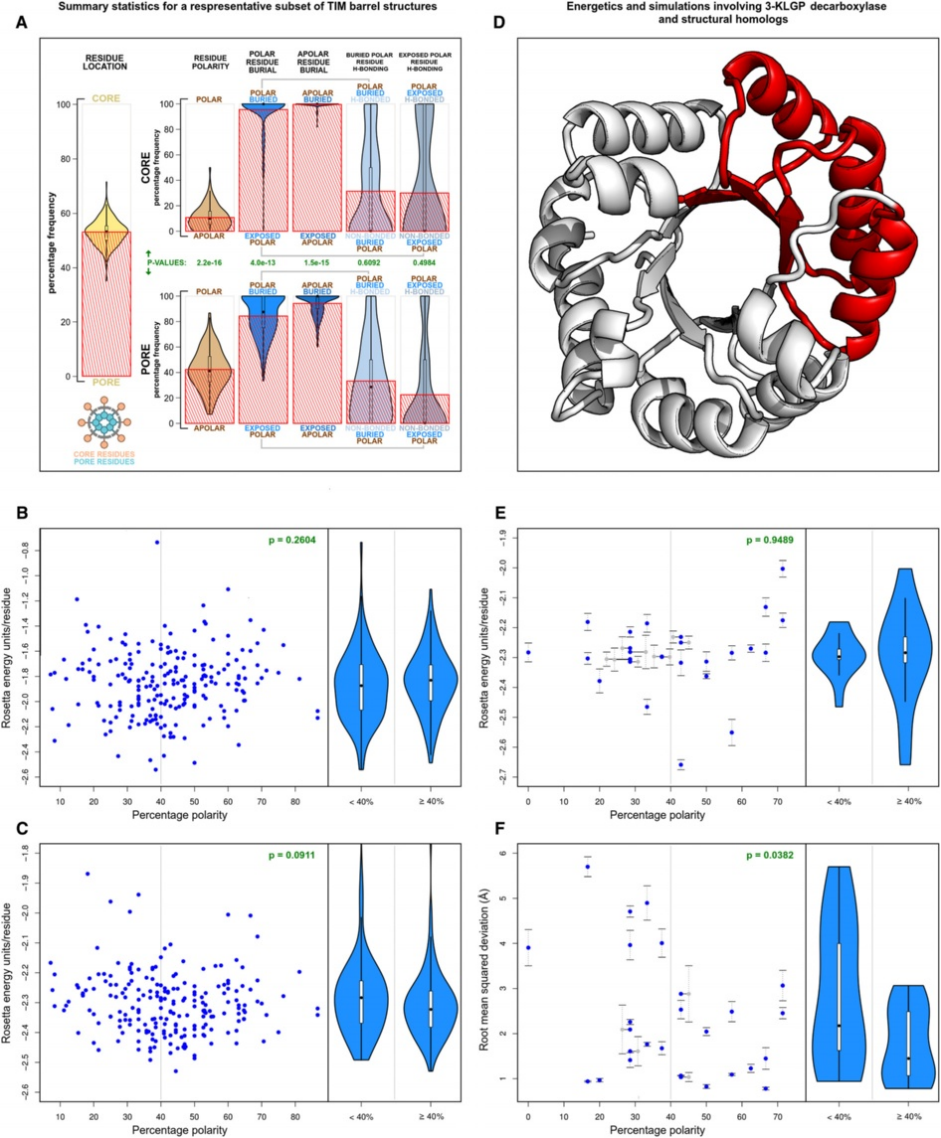
Summary statistics, energetics, and folding simulations for natural TIM barrels. **a** Summary statistics for a representative subset of TIM barrel structures derived from the CATH database. These statistics describe the residue composition and hydrogen bonding frequencies across different regions of the TIM barrel scaffold. Violin plots are used to visualize data distribution, while superimposed red histograms are used to visualize means. All p-values are calculated using the Welch two sample t-test. **b**,**c**,**e**,**f** General figure description: *Right*: Scatterplots describe the data distribution. A grey line divides the data into hydrophobic pores (<40 % polarity) and hydrophilic pores (≥40 % polarity). P values are depicted in green. All p-values are calculated using the Welch two sample t-test. *Left:* Violin plots depict the data distribution across hydrophobic and hydrophilic pores. **b** Variation of Rosetta energy scores against percentage pore polarity across all selected TIM barrel pores. Only pore residues are scored, and backbone hydrogen bonding terms are not included. **c** Variation of Rosetta energy scores against percentage pore polarity across all selected TIM barrel pores. Entire TIM barrel proteins are scored, and backbone hydrogen bonding terms are included. **d** The TIM barrel enzyme KLGP decarboxylase (PDB ID: 1kv8, chain A) is shown in white. The (αβ)_3_ foldon-like module chosen is shown in red. **e** Variation of Rosetta energy scores against percentage pore polarity for all selected (αβ)_3_ foldon-like modules of KLGP decarboxylase structural homologues. **f** Variation of the mean backbone RMSD (for the 5 ab initio modeled structures with the lowest Cα RMSD, compared to their (αβ)_3_ templates) against percentage pore polarity. This figure supports the application of the foldon hypothesis to TIM barrel proteins

In order to understand the functional significance of pore residues, we performed a statistical and energetic analysis on a 215-member representative subset of all TIM barrel structures from the CATH database. Beta-barrel residues were classified as belonging to the pore or core, and trends in residue nature, location, burial, and hydrogen bonding were noted. Summary statistics are presented in Fig. 3a. Beta-barrel residues were found to be evenly divided amongst the pore and core, with a mean pore residue prevalence of 52.96 %. Core beta-barrel residues were analyzed separately from the pore, acting as a proxy for the entire core region. Core and pore statistics were compared using the Welch two sample t-test. As expected, polar residues (excluding tyrosine and tryptophan. Glycine was not counted) were found to be significantly more prevalent within the pore. The mean pore residue polarity was found to be 42.38 %, eclipsing the mean core residue polarity of 10.78 % (p = 2.2e-16). The Naccess program was used to predict residue burial, and residues were classified as buried if their accessible surface areas were less than 30 % of the total. Pore residues were found to be significantly more exposed to solvent than their core counterparts. The mean polar pore residue burial was 84.28 %, lower than the mean polar core residue burial of 95.59 % (p = 4.0e-13). This trend was also observed for apolar residues (p = 1.5e-15). These trends were expected, as the pore is partially solvent exposed at its N-terminal and C-terminal faces. Hydrogen bonding patterns, however, showed counterintuitive trends. Hydrogen bonds were estimated using the Rosetta scoring function on all protein structures. Both polar buried and polar exposed residues inside the pore showed no statistically significant increase in hydrogen bonding as compared to their core counterparts. On average, 33.23 % of buried polar residues within the pore formed hydrogen bonds, as compared to 31.41 % of buried polar residues within the core (p = 0.6092). These trends were similar for exposed polar residues (p = 0.4984).

Since polar pore and polar core residues have statistically indistinguishable hydrogen bonding frequencies, their relative abundance within the pore region cannot be explained based on hydrogen bonding patterns alone. All energetic terms, including hydrogen bonding energies, the Lennard-Jones potential, the Lazaridis-Karplus solvation energy, rotamer energies calculated from Dunbrack's library, and Ramachandran-based backbone energies were taken into account while calculating the Rosetta score. We then attempted to determine the effect of pore residue polarity on the Rosetta energy score, and hence TIM barrel stability. Our analysis involved comparing the mean pore Rosetta energy score with percentage pore polarity (Fig. 3b). In this particular instance, backbone hydrogen bonding potentials were ignored, as pore residues are non-contiguous in sequence space. Hydrophobic and hydrophilic pore statistics were compared using the Welch two sample t-test. Once again, counterintuitive trends were observed. No statistically significant difference was observed when the scores of hydrophobic pores (<40 % polarity) were compared with scores of hydrophilic pores (≥40 % polarity) (p = 0.2604). This observation was confirmed for entire TIM barrel structures as well (Fig. 3c). No statistically significant difference was observed when the scores of structures containing hydrophobic pores were compared with scores of structures containing hydrophilic pores (p = 0.0911).

Since the abundance of polar pore residues could not be explained based on energetic terms, we attempted to explain their abundance in kinetic terms. Protein folding is a complex process, often involving the generation and disassembly of multiple transition states along a rough energy landscape. The *foldon* hypothesis [47, 48] describes the process of successful folding as one involving the progressive accumulation of correctly folded small secondary structure elements, or foldons, till the native structure is reached. The foldon hypothesis has been experimentally confirmed for small proteins including cytochrome c [47], RNase H [49], and apocytochrome b_562_ [50]. A recently study on the folding mechanism of a TIM barrel protein (*S. solfataricus* indole-3-glycerol phosphate synthase) has invoked the foldon hypothesis [51]. A conserved foldon-like βαβαβ module was described as an essential template, upon which other secondary structure elements folded. Beta barrel closure was described as the final step in folding. It is easy to see the role that polar pore residues would play in the stabilization of such a foldon. Local αβ modules could form early into the folding process, acting as nuclei for the progressive formation of other modules. These modules, including their pore residues, would be solvent-exposed, as beta barrel formation would not yet have occurred. At this stage in the folding process, polar pore residues would be energetically and evolutionarily favored. In order to determine whether the foldon hypothesis explained polar pore residue abundance, we performed folding simulations on a small, (αβ)_3_ foldon-like module derived from the 3-keto-L-gulonate 6-phosphate decarboxylase [52] (KLGP decarboxylase) (PDB ID: 1kv8, chain A) (Fig. 3d) and its structural homologues. KLGP decarboxylase was chosen for these folding simulations due to its simple structure and its maintenance of canonical TIM barrel topology. The eight helices and eight strands are connected by short, featureless loops. C-terminal domains are usually absent along the chosen module, and are restricted to short helices along the rest of the structure. KLGP decarboxylase structural homologues were identified from structures within the CATH database. All TIM barrel structures with a backbone RMSD of less than or equal to 3 Å were selected using Mustang (version 3.2.1) [53], and were filtered through a 90 % sequence identity threshold using CD-hit. KLGP decarboxylase and 26 structural homologues were selected, and all homologous (αβ)_3_ modules were identified. The Rosetta energy score and percentage pore polarity of these modules was calculated within their parent TIM barrel structures (Fig. 3e). In agreement with previous observations, no statistically significant difference was observed when the scores of modules containing hydrophobic pores were compared with scores of modules containing hydrophilic pores (p = 0.9489). This confirmed that polar pore residues within (αβ)_3_ modules play no energetic role in the native structures of KLGP decarboxylase structural homologues. Folding simulations, however, revealed the importance of polar pore residues for (αβ)_3_ module stabilization (Fig. 3f). The sequences of all 27 modules were extracted from their parent TIM barrel structures, and used for Rosetta ab initio modeling simulations. 2000 simulations were performed for each sequence. For each sequence, 5 modeled structures with the lowest Cα RMSD compared with their respective template modules were selected, and their mean RMSD was calculated. In agreement with the foldon hypothesis, the mean RMSDs of (αβ)_3_ modules containing hydrophilic pores showed a statistically significant decrease when compared to modules containing hydrophobic pores (p = 0.0382). The structures for all 27 modules are provided in the supporting information (Additional file 6: Dataset S1). A brief analysis of the ability of Rosetta to model naturally exposed hydrophobic patches is provided in the supporting information (Additional file 7: Text S3, Additional file 8: Dataset S2).

Energetic analyses and ab initio modeling results indicate that TIM barrel proteins containing polar pore residues have engaged in a trade-off between structural stability and foldability. Buried polar pore residues, despite their poor hydrogen bonding, aid in the folding process by stabilizing foldon modules. Without the aid of independently folding and progressively accumulating modules, the speed and efficiency of TIM barrel folding pathways containing polar pore residues would likely drastically decrease. It is worth mentioning that the foldon hypothesis cannot explain the folding mechanisms of TIM barrel proteins containing mostly hydrophobic pores. In these cases, folding may occur through multiple, evolutionarily-optimized transition states. Ultimately, the evolutionary preference for polar pore residues indicates the superiority of folding along foldon-like modules.

### Insights derived from designed TIM barrel structures

Early attempts at TIM barrel design included the proteins Octarellin I,II and III [20–22]. Octarellin I was designed using a sequence-based design protocol, based on principles derived from a limited number of TIM barrel structures. Octarellin I showed encouraging characteristics for an early designed protein, including an alpha-beta secondary structure, as determined by infrared spectroscopy. It also displayed stability on thermal denaturation. Later, an improved design methodology incorporated improvements in charge distribution, incorporated discreet hydrophobicity and volume criteria, and included data from an expanding library of TIM barrel structures. This methodology was tested in the Octarellin II and Octarellin III designs. CD spectroscopy revealed an alpha-beta secondary structure for both proteins. Octarellin III appeared more stable, and showed cooperative unfolding characteristics on urea denaturation.

With increasing computational resources, structure-based design using all-atom models became possible. Attempts to design TIM barrels based on improved algorithms resulted in the creation of Octarellin V [23] and Octarellin VI [24]. For Octarellin V, the ORBIT (optimization of rotamers by iterative technique) program [2] was used during the design process. Improved search algorithms based on the dead-end elimination method were used to search side-chain conformational space. A potential energy function ranked sequences based on the solvent accessibility, the van der Waals potential, a solvation potential, and hydrogen bonding potentials. Octarellin V displayed greater stability to denaturation and improved cooperative characteristics, as determined by guanidine hyrochloride (GuHCl) unfolding experiments. Although broad peaks and low dispersion were observed during 1D-NMR experiments, the protein still appeared to adopt some tertiary structure. The most recent design, Octarellin VI, was created using methods implemented in the Rosetta software suite. This design showed improvements in thermal stability.

The most encouraging trait displayed in previously designed TIM barrel structures was their cooperative unfolding, observed under urea and guanidine hydrochloride denaturing gradients.

Natural, well ordered proteins are distinguished by cooperative characteristics [54]. Experimentally, cooperativity in proteins is characterized by an abrupt, sigmoidal transition in state, detected as a function of temperature or denaturant. Cooperative folding in proteins is likely a result of natural selection, as it increases protein stability in vivo and prevents detrimental aggregation [55]. Unfortunately, all designs displaying cooperative unfolding also showed poor solubility and a tendency to aggregate. For experimental characterization, these designs were extracted and refolded from inclusion bodies.

An analysis of Octarellin sequences revealed a possible reason for their insolubility and tendency to aggregate. All sequences show low percentages of polar pore residues. The percentage pore polarities of Octarellin I, II, III, V, and VI are 0 %, 25 %, 22.22 %, 0 %, and 17.64 % respectively. As previously described, the mean pore polarity of a representative subset of TIM barrel structures was found to be 42.38 %, and polar pore residues were shown to play a vital role in protein folding through their stabilization of foldon-like modules. Although natural TIM barrels with low percentage pore polarities exist, they are products of evolution and natural selection. Such TIM barrels would likely cooperatively fold through complex, evolutionarily-optimized pathways involving multiple transition states. The creation of such pathways is beyond the capabilities of current protein design technology, with even simple designs showing non-optimal folding characteristics [56, 57]. Therefore, given the current state-of-the-art in protein design technology, the design of TIM barrels with apolar pores would likely be extremely difficult.

We further investigated the ability of later Octarellin designs to fold along independent modules.

**Fig. 4:**
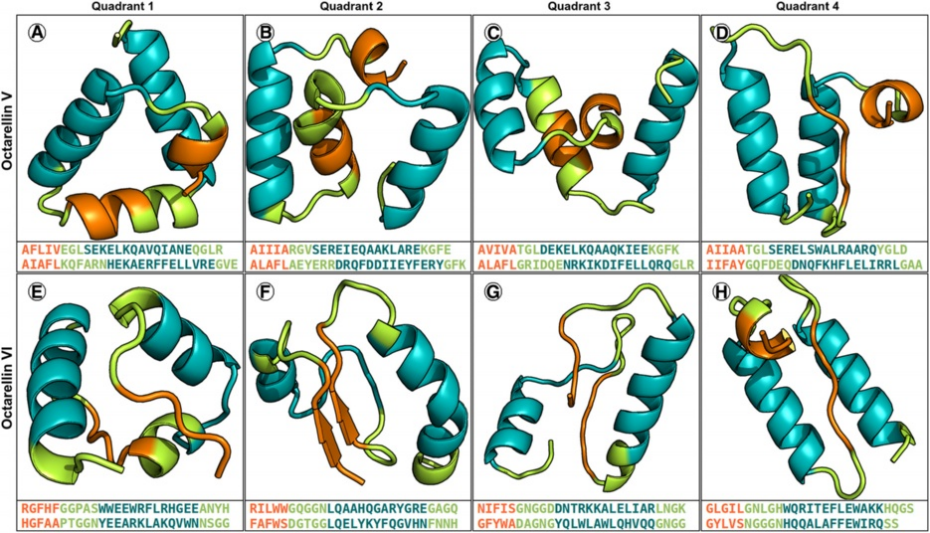
Ab initio modeling of Octarellin V and Octarellin VI. Both sequences were divided into four quadrants each before ab initio modeling. **a**-**d** The lowest scoring modeled structures for all fragments of Octarellin V, arranged from N to C-terminal. The expected alpha/beta secondary structure topology is not observed. Instead, all fragments adopt mostly helical secondary structures. **e**-**h** The lowest scoring modeled structures for all fragments of Octarellin VI, arranged from N to C-terminal. Although some beta sheets are observed, the overall topology is inconsistent with that expected for a TIM barrel. Note that the intended design of secondary structure is depicted in colors. Designed alpha helices are colored teal. Designed beta sheets are colored orange. Designed loops are colored green

The sequences of Octarellin V and VI were divided into 4 (αβ)_2_ modules each. The sequences for these 8 (αβ)_2_ modules were each subjected to 2000 iterations of Rosetta ab initio folding, and the secondary structure and topology of the lowest scoring structures were observed for each module. In all 8 cases, the modeled secondary structure and topology of the lowest scoring ab initio structures differed from their intended design (Fig. 4). For Octarellin V, sequences designed as beta sheets were modeled as helices (Fig. 4a-d). These results indicate that beta sheets constituting a completely hydrophobic pore are unstable in solvent-exposed modules, and consequently collapse into helices. For Octarellin VI, sequences designed as beta strands were mostly modeled as beta strands or loops (Fig. 4e-h). However, no model adopted the correct secondary structure and topology. Furthermore, for the 2000 models generated for each module, no structural consensus was found amongst the lowest scoring models.

In order to eliminate possible bias originating from the use of Rosetta for both design as well as analysis, we performed the same modeling experiments using the QUARK ab initio modeling tool [58]. QUARK was chosen for its successful performance in the CASP experiments [59], as well as its successful use in a recent study involving experimental protein redesign [60]. The ab initio modeling results generated by QUARK were similar to those of Rosetta. Octarellin V was poorly modeled, with (αβ)_2_ modules modeled as helical bundles. Although still imperfect, Octarellin VI was comparatively better modeled, with two out of four (αβ)_2_ modules displaying a somewhat correct topology. The best QUARK ab initio models are illustrated in the supporting information (Additional file 9: Figure S2), and all QUARK ab initio models are provided in the supporting information (Additional file 10: Dataset S3) .Therefore, these results indicate that later Octarellin designs do not fold into their designed topologies, and may adopt an ensemble of structural states.

### Computational design of an ideal, symmetric TIM barrel protein

**Fig. 5:**
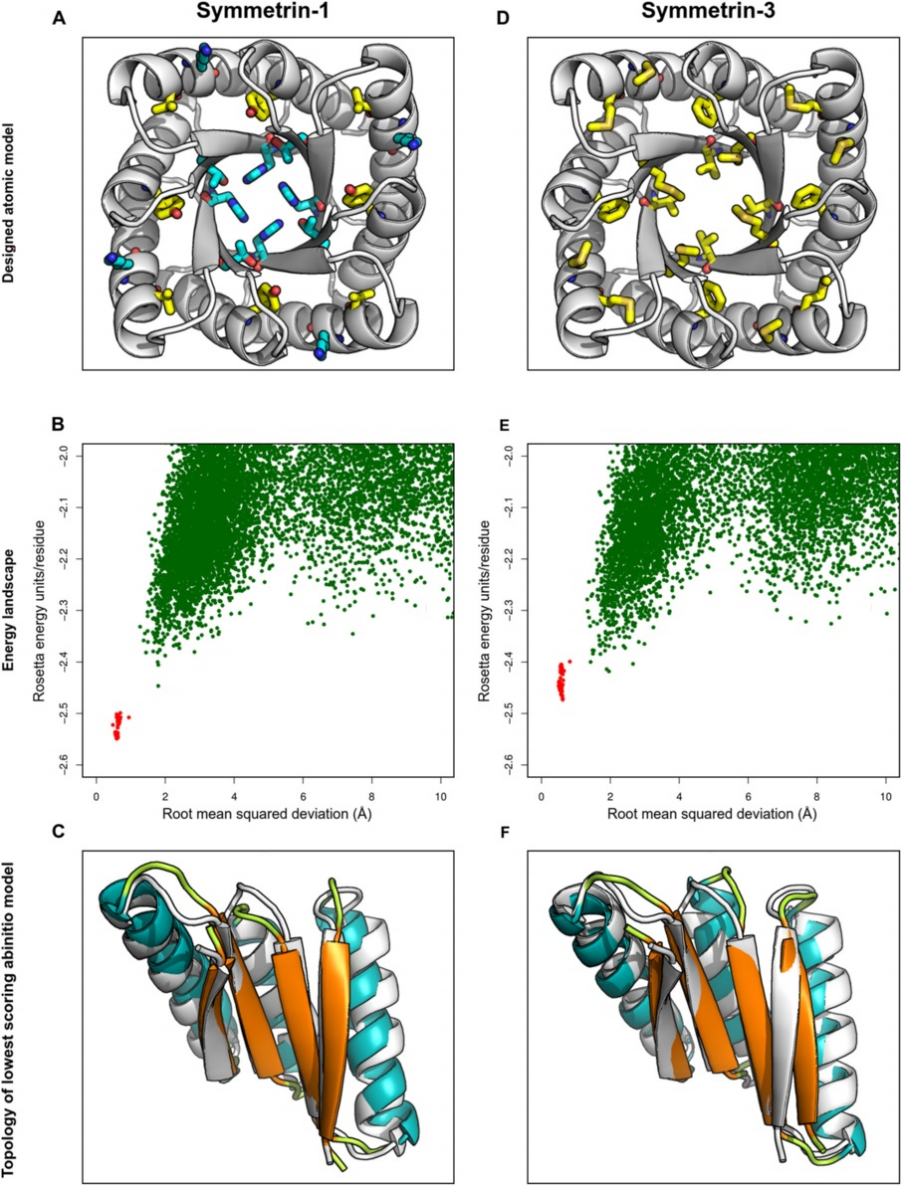
Folding landscape of Symmetrin-1 and Symmetrin-3. **a** The Symmetrin-1 backbone model is shown in white. All residues mutated in Symmetrin-3 are colored. Hydrophobic residues are colored yellow. Hydrophilic residues are colored cyan. **b** A Cα root mean squared deviation (RMSD) vs. Rosetta energy units/residue plot for a 95-residue α_3_β_4_ module of Symmetrin-1, representing all designed inter-residue interactions. A funneled folding landscape is apparent. 10,000 ab initio modeling iterations were performed, depicted as green dots. 100 all-atom energy minimizations of the 95-residue fragment were performed, depicted as red dots. **c** Superposition of the designed 95-residue Symmetrin-1 α_3_β_4_ module (white) upon the lowest scoring ab initio model (colored by secondary structure). The secondary structural elements and overall topology is preserved. **d** The Symmetrin-3 backbone model is shown in white. All residues differing from Symmetrin-1 are colored. Hydrophobic residues are colored yellow. Hydrophilic residues are colored cyan. **e** A Cα root mean squared deviation (RMSD) vs. Rosetta energy units/residue plot for a 95-residue α_3_β_4_ module of Symmetrin-3, representing all designed inter-residue interactions. A funneled folding landscape is apparent, although funneling is slightly less prominent as compared to Symmetrin-1. 10,000 ab initio modeling iterations were performed, depicted as green dots. 100 all-atom energy minimizations of the 95-residue fragment were performed, depicted as red dots. **f** Superposition of the designed 95-residue Symmetrin-3 α_3_β_4_ module (white) upon the lowest scoring ab initio model (colored by secondary structure). The secondary structural elements and overall topology is preserved

Insights derived from existing TIM barrel structures and previous TIM barrel design attempts were applied for the computational design of a new, symmetric TIM barrel protein. An ideal TIM barrel scaffold was assembled from protein fragments based on geometrical and topological first principles. Sequences were designed for the scaffold through a design methodology that iterated through fixed backbone sequence design, symmetry enforcement, and all-atom relaxation protocols. 1000 structures were designed, and a low scoring sequence whose correctly folded structure occupied the global energy minima was chosen. This design protocol yielded a 224-residue structure named Symmetrin-1, containing 56-residue 4-fold symmetric repeats (Figs. 1a-b, 5a). This structure was selected for variant generation, gene-synthesis, and experimental characterization.

The computational model of Symmetrin-1 appeared well ordered, preserving the secondary structure and backbone symmetry of the initial designed scaffold. The structure possessed a low Rosetta energy score under standard parameters, scoring −634.257 R.E.U in total and −2.83 R.E.U/residue. The pore of Symmetrin-1 was populated with polar residues required for modular folding and was characterized by an extensive hydrogen bonding network dominated by histidine residues (Fig. 5a, Additional file 11: Figure S3). Ab initio modeling was performed for 10,000 iterations, generating a well funneled folding landscape (Fig. 5b). These results were confirmed using QUARK (Additional file 9: Figure S2I). These results predicted that the designed sequence adopted the correct structure and occupied the global energy minima. No alternate energetically favorable conformations were observed. The lowest scoring ab initio model possessed the desired secondary structure and topology (Fig. 5c). Furthermore, the Cα RMSD between the designed structure and ab initio model was found to be 1.8 Å.

The sequence of Symmetrin-1 was unique, as determined by a BLAST search [61] across known protein sequences in the NCBI database. No significantly similar sequences were found for the 56-residue symmetric repeat. The closest similarity detected was for the UPF0597 protein from Prevotella denticola (NCBI ID: 767031), with an E-value of 0.28.

Three Symmetrin-1 variants were created, primarily differing in pore residue composition. Symmetrin-2 was designed with a single H(116) → W mutation (Additional file 11: Figure S3), in order to determine if histidine-tryptophan aromatic interactions [62–64] could confer additional stability to the structure. Symmetrin-3 (Fig. 5d-f, Additional file 9: Figure S2J, Additional file 11: Figure S3) and Symmetrin-4 (Additional file 11: Figure S3) were designed with slightly more hydrophobic pores, in order to explore the trade-off between stability and foldability previously described. The sequences, structures, and resfiles of all Symmetrin designs are provided in the supporting information (Additional file 12: Dataset S4).

### Experimental characterization of computational designs

The genes for all Symmetrin designs were synthesized, and their corresponding proteins were expressed and experimentally characterized. A summary of all experimental results is presented in Table 1. Of the 4 proteins designed and tested, Symmetrin-1 and Symmetrin-3 displayed the most noteworthy characteristics, and have been described in detail below. Symmetrin-2 displayed identical spectral, thermal, and gel filtration characteristics as compared to Symmetrin-1, and was not tested further. Symmetrin-4 was insoluble, and could not be tested further.

**Table 1:** Experimental characterization of Symmetrin proteins. Each column displays the results of a single experiment performed on all Symmetrin designs. Each row displays the results of all experiments performed on a single Symmetrin design. The Symmetrin-1 and Symmetrin-3 designs displayed the most noteworthy characteristics. Cells left blank represent experiments not performed

Protein	Expression	Solubility	CD spectrum	T_m_	FPLC	1D NMR
Symmetrin-1	10 mg/l	soluble	alpha-beta	44 °C	monomeric	well resolved
Symmetrin-2	10 mg/l	soluble	alpha-beta	44 °C	monomeric	
Symmetrin-3	10 mg/l	soluble	alpha-beta	63 °C	oligomeric	poorly-resolved
Symmetrin-4	3 mg/l	insoluble				

**Fig. 6:**
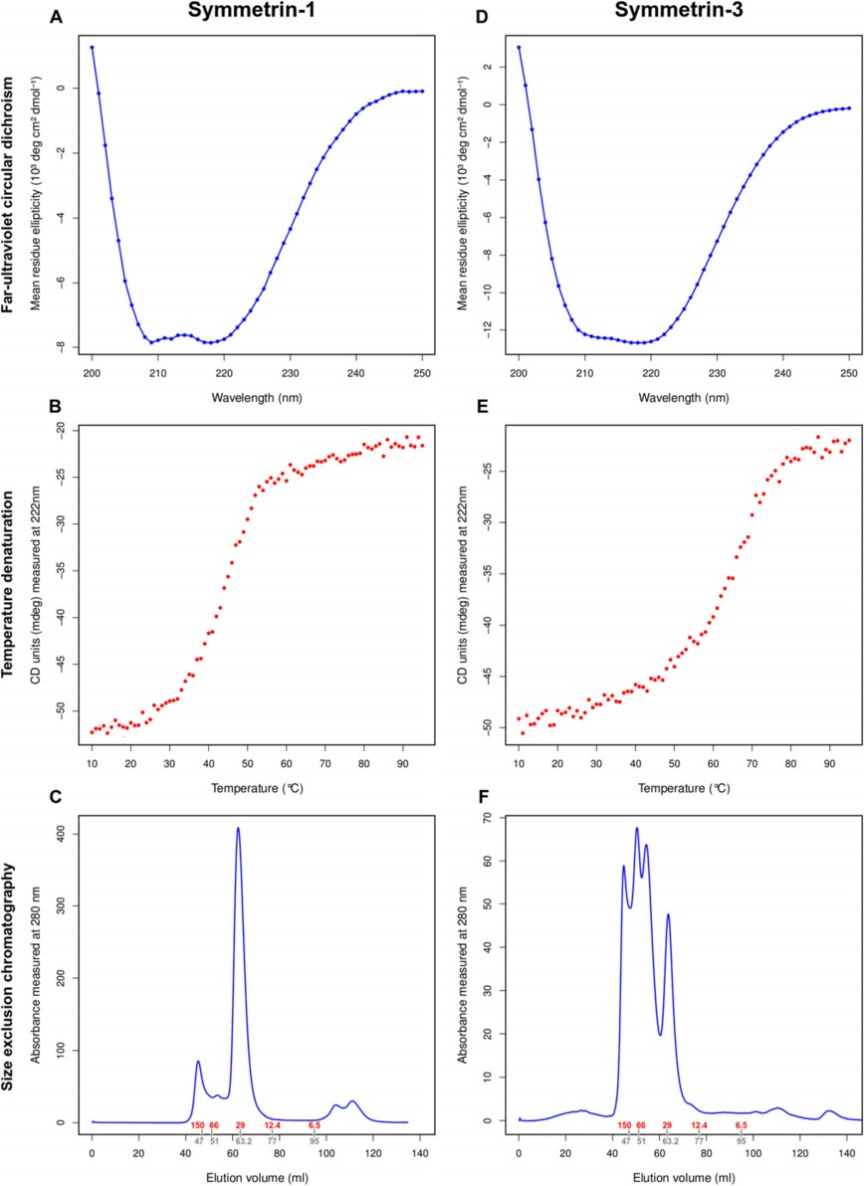
Experimental characterization of Symmetrin-1 and Symmetrin-3. Figures (**a**,**b**,**c**) show experimental results for Symmetrin-1. **a** The far-ultraviolet circular dichroism (CD) spectrum is shown, displaying prominent alpha-beta characteristics. **b** Temperature denaturation recorded at 222 nm from 10-95 °C. The denaturation curve appears sigmoidal. **c** Gel filtration (FPLC) data, showing a prominent peak at 62.43 ml. Elution volumes for markers are shown in grey, while their corresponding molecular weights are shown in red. Figures (**d**,**e**,**f**) show experimental results for Symmetrin-3. **d** The far-ultraviolet CD spectrum is shown, displaying prominent alpha-beta characteristics. **e** Temperature denaturation recorded at 222 nm from 10-95 °C. The denaturation curve appears sigmoidal. Note that the protein appears more stable than Symmetrin-1. **f** Gel filtration (FPLC) data, showing a many peaks from 50.38 ml to 64.18 ml. Elution volumes for markers are shown in grey, while their corresponding molecular weights are shown in red

Symmetrin-1 was easily expressed, with cultures yielding 10 mg/l protein on IPTG induction. The protein was found to be readily soluble, and was stored at 4 °C at concentrations of 2.5 mg/ml. Circular dichroism experiments were performed to determine the secondary structure content and stability of Symmetrin-1 under different denaturing conditions. The far UV CD spectrum revealed that Symmetrin-1 adopts a secondary structure consistent with alpha/beta proteins, showing two characteristic minima near 220 nm and 210 nm (Fig. 6a).

Temperature denaturation experiments were performed by observing changes in the far UV CD spectrum measured at 222 nm. The change in signal strength through a temperature gradient of 10-95 °C was smooth and sigmoidal, indicating cooperative unfolding (Fig. 6b). Thermodynamic analyses were performed by fitting the transition region of the temperature denaturation curve into the Van't Hoff equation. From these calculations, the melting temperature (T_m_) was determined to be 44.0 °C, and the Gibbs free energy of unfolding was determined to be 8.0 kJ/mol.

Upon gel filtration (Fig. 6c), a single peak was observed at an elution volume of 62.43 ml. Using protein standards (Additional file 13: Figure S4) , the molecular weight of this peak was estimated to be 32.12 kDa, in fair agreement with a theoretical molecular weight of 25902.77 Da, calculated using the ExPASy webserver [65]. A peak corresponding to the void volume elution was detected at 45.27 ml. Minor peaks were detected between 100 ml and 120 ml. A SDS-PAGE analysis of all eluted peaks revealed that Symmetrin-1 was restricted solely to the major peak eluted at 62.43 ml. These results indicated that Symmetrin-1 is monomeric in solution, and does not aggregate on storage.

Symmetrin-3 showed similar expression and solubility characteristics as Symmetrin-1, despite an increase in the number of apolar residues. Cultures yielded 10 mg/l protein on IPTG induction, and the protein was stored at 4 °C at concentrations of 2.5 mg/ml. The far UV CD spectrum of Symmetrin-3 was similar to that of Symmetrin-1, revealing an alpha/beta secondary structure, with characteristic minima near 220 nm and 210 nm (Fig. 6d).

Temperature denaturation experiments revealed noteworthy differences between the energetics of Symmetrin-3 and Symmetrin-1. For Symmetrin-3, the change in signal strength through a temperature gradient of 10-95 °C once again indicated cooperative unfolding (Fig. 6e). Thermodynamic analyses revealed that Symmetrin-3 displays superior thermal stability characteristics. Upon fitting the transition region of the temperature denaturation curve into the Van't Hoff equation, the T_m_ was determined to be 63.0 °C, representing a 19 °C improvement over Symmetrin-1. Furthermore, the Gibbs free energy of unfolding was determined to be 10.6 kJ/mol, representing a 2.6 kJ/mol improvement over Symmetrin-1. Although these results are encouraging, they may be a result of oligomerization, as described below.

Gel filtration (Fig. 6f), however, revealed inferior characteristics for Symmetrin-3. Unlike Symmetrin-1, Symmetrin-3 was found to display three peaks eluted at 64.18 ml, 54.29 ml, and 50.38 ml. Based on apparent molecular weights calculated from protein standards, these three peaks were determined to be monomeric(27.30 kDa), dimeric(52.86 kDa), and trimeric(75.01 kDa) forms respectively. A peak detected at 44.78 ml corresponded to the void volume elution. A SDS-PAGE analysis of all eluted peaks revealed that the monomeric peak was composed exclusively of Symmetrin-3, whereas the dimeric and trimeric peaks contained Symmetrin-3 as the majority component along with varying degrees of impurities. The void volume elution was also found to contain Symmetrin-3 as a minor component. These results indicate that Symmetrin-3 oligomerizes in solution. Furthermore, the isolated monomeric species appeared to slowly convert to oligomeric forms upon prolonged storage (Additional file 14: Figure S5). Despite its tendency to oligomerize, the apparent molecular weight of the monomeric peak (27.30 kDa) indicated that it may be relatively more compactly folded than the Symmetrin-1 monomer, and is in good agreement with the theoretical molecular weight of 25947.41 Da, calculated using the ExPASy webserver [65].

### Structural insights through NMR

**Fig. 7:**
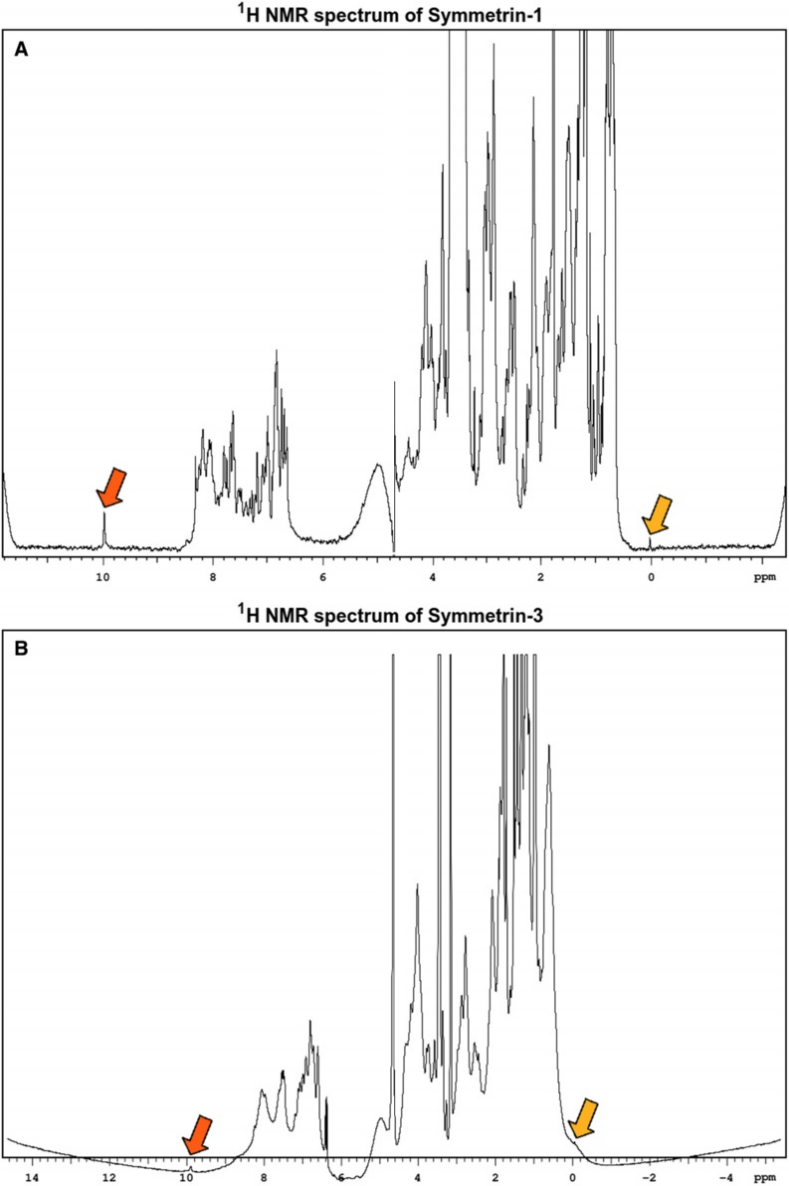
1D-NMR spectrum of Symmetrin-1 and Symmetrin-3. **a** 1D-NMR spectrum for Symmetrin-1. Sharp, prominent chemical shifts are observed. However, dispersion is rather limited. **b** 1D-NMR spectrum for Symmetrin-3. Less prominent chemical shifts were observed. In both figures, the methyl chemical shift at 0.0 ppm, and the tryptophan chemical shift at 10.0 ppm, are highlighted using yellow and orange arrows, respectively

The 1D-NMR spectrum of Symmetrin-1 appeared well resolved, with sharp chemical shifts found within a range of 0-10 ppm (Fig. 7a). In a well folded protein, chemical shifts downfield of the amide region (>8.5 ppm) and within the methyl region (<0.5 ppm) are expected. For proteins with aromatic residues, additional downfield dispersion (9.5-11.5 ppm) is expected. In the case of Symmetrin-1, a strong chemical shift caused by Nε atoms was observed at 10 ppm, and reflected the presence of tryptophan residues in the structure. A small but sharp chemical shift at 0.0 ppm occupied the methyl region, indicating ordered methyl groups. Excluding these chemical shifts, sparse dispersion was observed in these regions.

**Fig. 8:**
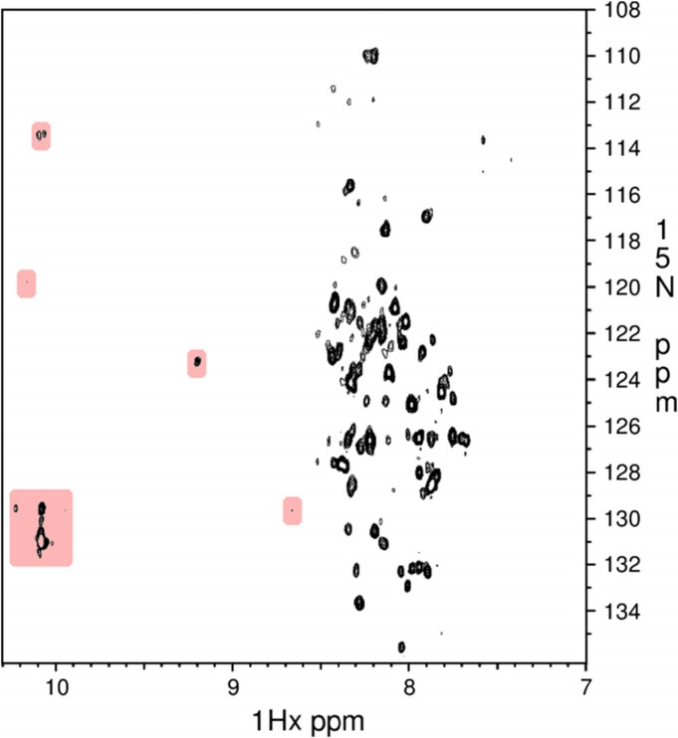
^15^N HSQC spectrum for Symmetrin-1. Dispersion beyond 8.5 ppm (^1^H-X axis) is highlighted in pink

2D HSQC experiments were performed using ^15^ N-labelled Symmetrin-1 (Fig. 8). The spectrum displayed well resolved peaks with little overlap, indicating the absence of aggregates and completely unstructured elements. However, the spectrum shows little dispersion beyond 8.5 ppm (^1^H-X axis). Such sparse dispersion is indicative of a lack of stable structural elements. These results indicate that Symmetrin-1 adopts a somewhat molten state in solution.

The monomeric peak obtained from gel filtration was used for acquiring the 1D NMR spectrum of Symmetrin-3. In contrast to Symmetrin-1, Symmetrin-3 displayed a narrower chemical shift dispersion with broader, smaller peaks (Fig. 7b). This is especially apparent for the Nε chemical shift (10.0 ppm) and the methyl chemical shift (0.0 ppm). However, slightly better dispersion was observed upfield in the methyl region and downfield in the amide region. Although insufficiently resolved, chemical shifts extended to −0.4 ppm upfield and 8.7 ppm downfield. Interestingly, resonances (presumably Hα protons) can be observed in the chemical shift region between 5–6 ppm. In general, these downfield shifted Hα protons are observed in proteins which harbor beta pleated strands as a secondary structure element [66, 67].

### Additional experimental characterization of Symmetrin-1

Spectrophotometric and gel filtration experiments confirmed the cooperativity and monomeric nature of Symmetrin-1. However, NMR experiments suggested that Symmetrin-1 assumes a somewhat molten state. The observation of this state implies a failure of rigid packing between side chains. However, molten globules undergo hydrophobic collapse, and Symmetrin-1 may retain the overall designed topology, albeit without well defined side chain packing. We performed additional experiments to confirm the stability of this globular state, and to further examine the cooperative characteristics observed during thermal denaturation.

**Fig. 9:**
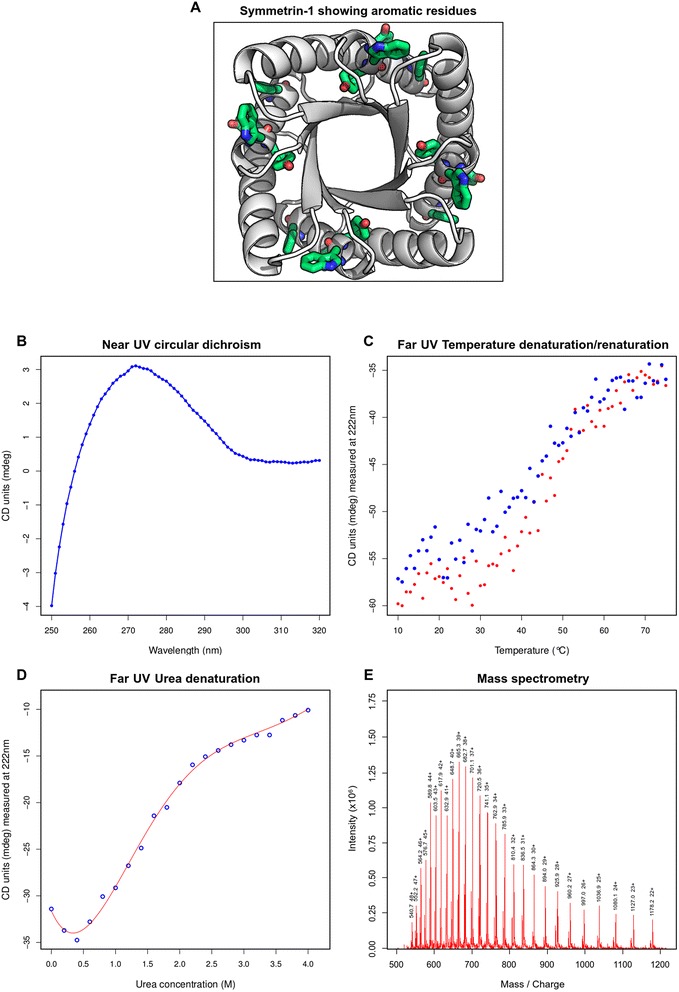
Additional experimental characterization of Symmetrin-1. **a** The Symmetrin-1 backbone model is shown in white. All aromatic residues are colored green. **b** The near-ultraviolet circular dichroism (CD) spectrum is shown, signifying immobilized aromatic residues. **c** Thermal unfolding and refolding assay, recorded at 222 nm from 10-75 °C. Unfolding data (heating schedule) is shown in red. Refolding data (cooling schedule) is shown in blue. **d** Chemical denaturation recorded at 222 nm from 0-4 M Urea concentration. The red line of best fit was generated from fifth-degree polynomial fitting and is intended as a guide to the eye. **e** LC-MS data, confirming the molecular weight of Symmetrin-1

Symmetrin-1 contains four phenylalanine, eight tyrosine, and four tryptophan residues (Fig. 9a). The presence of aromatic residues allowed us to gauge hydrophobic collapse through the observation of the near UV CD spectrum, which is typically dominated by contributions from immobilized aromatics. The near UV CD spectrum displayed a strong positive signal, with a maxima located at 275 nm (Fig. 9b). This signal ranged from 255 nm to 300 nm, and is sufficiently dispersed to include contributions from all phenylalanine, tyrosine, and tryptophan residues in their buried states.

In order to understand the refolding dynamics of Symmetrin-1, a thermal unfolding and refolding assay was performed (Fig. 9c). This experiment was performed by observing changes in the far UV CD spectrum measured at 222 nm. A temperature gradient of 10-75 °C was selected, corresponding to the transition region previously determined for Symmetrin-1. As expected, during the heating schedule (10 → 75 °C), the observed transition was smooth and sigmoidal, indicating cooperative unfolding. During the cooling schedule (75 → 10 °C), we observed that while refolding, Symmetrin-1 retraced the path of its sigmoidal unfolding transition. These observations demonstrate the reversible thermal unfolding properties of Symmetrin-1. Such properties are essential to natural proteins, enabling them to recover their structure quickly after perturbation.

In order to confirm the Gibbs free energy of unfolding calculated from thermal denaturation, chemical denaturation was performed using a gradient of 0 M-4 M urea (Fig. 9d). After an initial negatively sloping baseline, higher urea concentrations caused a smooth and sigmoidal change in signal strength, indicating cooperative unfolding. Using methods previously described, the melting concentration (C_m_) was determined to be 1.6 M urea, and the Gibbs free energy of unfolding was calculated to be 8.3 kJ/mol, in good agreement with the value determined by temperature denaturation (8.0 kJ/mol).

A confirmation of the mass of Symmetrin-1 was performed using liquid chromatography-mass spectrometry (LC-MS) (Fig. 9e). The majority species was found to possess a molecular weight of 25905.7 Da, in very good agreement with the theoretical molecular weight.

## Conclusions

The Rosetta software suite has been successfully used in the de novo design of several small, 100-residue proteins [8, 9], and has been used to design symmetric protein complexes, composed of asymmetric protein subunits [19]. Inspired by these successes, we attempted to use Rosetta to design a 224-residue, 4-fold symmetric protein that adopts an ideal TIM barrel fold. Thus, we have designed, expressed, purified, and experimentally characterized the Symmetrin family of proteins.

This study aims to contribute to the advancement of TIM barrel design. Our biggest contributions are an increase in solubility, monomeric stability, and improved cooperative folding characteristics. We believe that the positive characteristics of Symmetrin-1 are attributable to an adherence to ideal topological rules during backbone construction. We believe that the adoption of the foldon hypothesis, and the design of polar pore residues, helped contribute to the reversible and cooperative foldability of Symmetrin-1, as observed during thermal renaturing experiments. Furthermore, we believe that the use of negative design features, involving the generation of folding landscapes for foldon-like modules, also contributed to the desirable folding characteristics of Symmetrin-1.

Symmetrin-1 displayed excellent solubility and folding characteristics, superior to those of any previously designed TIM barrel protein. However, NMR experiments revealed that it failed to adopt a well defined tertiary structure. Pore residue design is one possible reason for the observed molten nature. The designed native state of Symmetrin-1 relies on an intricate, histidine-dominated hydrogen bonding network for pore stability. While the formation of soluble, cooperatively folding, alpha-beta secondary structural units has been established, it is possible that the designed histidine-dominated hydrogen bonding network may be unstable, and therefore hinders the pore beta-barrel formation. This possibility is supported by the observations that the Rosetta software suite performs poorly when designing buried hydrogen bonds at protein interfaces [68].

Pore redesign attempts must make extensive use of polar residues. We have established that polar residues are required for proper foldon-like module formation. Symmetrin-3 (75 % pore polarity) and Symmetrin-4 (62.5 % pore polarity) were designed with relatively less polar pore residues, in order to explore the trade-off between foldability and stability. Although Symmetrin-3 showed a higher Gibbs free energy of unfolding, indicating better stability, it was found to oligomerize in solution, indicating poorer folding characteristics. Symmetrin-4 proved to be a remarkably poor design, and was found to be completely insoluble. All pores have been illustrated in Additional file 11: Figure S3. One possible redesign solution would involve the extensive use of asparagine and glutamine residues. Work on glutamine rich repeats has shown that glutamine residues can autonomously form extensive hydrogen bonding networks [69, 70], making them ideal pore residues.

We have computationally and experimentally studied the application of the foldon hypothesis to TIM barrel folding. However, our p-value (p = 0.0382) establishing statistical significance still leaves a margin for error, and our experimental characterization relied on a relatively small dataset of 4 proteins. Further large-scale computational and experimental work would therefore be required to exhaustively and unambiguously understand TIM barrel folding.

Although the Rosetta software suite represents the state of the art in computational protein design, its energy functions contain several assumptions and simplifications, reducing overall accuracy. Koga et al. reported a 20 % success rate during the large-scale design of small proteins using the Rosetta software suite [9]. However, we have demonstrated that the design of large proteins is possible using current protein design technology, and additional design attempts incorporating the suggested alterations may produce a well folded, stable, symmetric TIM barrel.

### Availability of supporting data

All supporting data for this article has been included with this article. All supporting data for this article is also available in the labarchives.com repository, and may be accessed via DOI as follows: Dataset S1: 10.6070/H4NC5Z69, Dataset S2: 10.6070/H4HM56FH, Dataset S3: 10.6070/H4833Q17, Dataset S4: 10.6070/H4833Q17, Figure S1: 10.6070/H44F1NQT, Figure S2: 10.6070/H40P0X1Z, Figure S3: 10.6070/H4VX0DJN, Figure S4: 10.6070/H4R49NS7, Figure S5: 10.6070/H4MC8X1G, Protocol S1: 10.6070/H4GM8594, Protocol S2: 10.6070/H4BV7DMV, Text S1: 10.6070/H4765C95, Text S2: 10.6070/H43F4MMW, Text S3: 10.6070/H4ZP4440. The plasmids for all proteins described may be purchased from Genewiz, Inc. by reference to their names and tracking numbers as follows: Symmetrin-1: 10–225266636, Symmetrin-2: "?>10–225266636, Symmetrin-3: 10–234215549, Symmetrin-4: 10–251299305.

## Additional files

Additional file 1:
**Text S1.** CATH identifiers for 215 non-redundant TIM barrel structures used in this study. (TXT 4 kb) Additional file 2:
**Protocol S1.** Scripts for a classification algorithm used to identify beta-barrel core or pore residues. (PDF 29 kb) Additional file 3: Figure S1. Intrastrand and interstrand distances have been described for two parallel beta strands. (PDF 67 kb) Additional file 4:
**Text S2.** The Cα-based model of the beta-barrel backbone used in this study. (CSV 2 kb) Additional file 5:
**Protocol S2. **Scripts for the positive design workflow required to design 4-fold symmetric TIM barrel proteins. (PDF 29 kb) Additional file 6:
**Dataset S1. **Structures for 27 (αβ)_3_ modules of KLGP decarboxylase structural homologues. These modules were used in ab initio folding simulations. (PDF 29 kb) Additional file 7:
**Text S3.** Modeling of exposed hydrophobic patches using Rosetta. (PDF 320 kb) Additional file 8:
**Dataset S2.** Structures for 13 (αβ)_3_ modules of proteins adopting the horseshoe fold. These modules were used in ab initio folding simulations. (PDF 29 kb) Additional file 9: Figure S2. Ab initio modeling of Octarellin V, Octarellin VI, and Symmetrin-1/3 using QUARK. All sequences were divided into four quadrants each before ab initio modeling. For each quadrant, the QUARK ab initio modeling tool generated 10 structures (provided within this dataset). Of the 10 models generated by QUARK, the model most closely resembling the designed topology is displayed. In cases where no topological resemblance was found, the first model is displayed. (A-D) Representative models for all fragments of Octarellin V, arranged from N to C-terminal. The expected alpha/beta secondary structure topology is not observed. Instead, most fragments adopt mostly helical secondary structures. For the fourth quadrant (D), although the correct topology is observed, beta sheets are modeled as disordered loops. (E-H) Representative models for all fragments of Octarellin VI, arranged from N to C-terminal. The second and third quadrants (F-G) display the correct topology. However, beta sheets are poorly modeled. Loops are overextended, cannibalizing residues designed as beta sheets. (I-J) Representative models for the 56-residue quadrants of Symmetrin-1 and Symmetrin-3. In both cases, the correct topology is observed. The ab initio models agree well with the designed structures (depicted in white), and a Kabsch superposition revealed low RMSD values (1.8 Å for Symmetrin-1 and 1.7 Å for Symmetrin-3). Note that designed secondary structure is depicted in colors. Designed alpha helices are colored teal. Designed beta sheets are colored orange. Designed loops are olored green. (PDF 1472 kb) Additional file 10:
**Dataset S3. **QUARK ab initio models for Octarellin V, Octarellin VI, Symmetrin-1, and Symmetrin-3. (PDF 29 kb) Additional file 11: Figure S3. (αβ)_2_ units are displayed for Symmetrin-1 to Symmetrin-4. We observed that protein stability decreases with decreasing pore polarity. Symmetrin-1 and Symmetrin-2 contain a percentage pore polarities of 100 % and 96.88 % respectively, and are the most stable designs. Symmetrin-3 contains a pore polarity of 75 %, and is relatively unstable, oligomerizing in solution. Symmetrin-4 contains the lowest pore polarity (62.5 %), and is completely insoluble. Hydrophobic residues are coloured yellow. Hydrophilic residues are coloured light teal. (PDF 73 kb) Additional file 12:
**Dataset S4.**Sequences, structures, and resfiles for all Symmetrin designs are provided. (PDF 29 kb) Additional file 13: Figure S4. Standard curve for the estimation of the molecular weight of Symmetrin-1 and Symmetrin-3. Proteins similar in molecular weight to Symmetrin-1 and Symmetrin-3 were considered while plotting the standard curve. Albumin (66 kDa), Carbonic Anhydrase (29 kDa), and Cytochrome C (12.4 kDa) were considered while creating the standard curve. (PDF 69 kb) Additional file 14: Figure S5. Size exclusion chromatography performed on isolated monomeric Symmetrin-3. Previously isolated monomeric Symmetrin-3 was stored at 4 °C/48 h prior to this experiment. The monomeric Symmetrin-3 species appears to slowly convert to oligomeric forms. (PDF 84 kb)
