# Supplementary material for: Design of symmetric TIM barrel proteins from first principles
Source: BMC Biochem. 2015 Aug 12;16:18. doi: 10.1186/s12858-015-0047-4 (PMC4531894; doi:10.1186/s12858-015-0047-4)
Supplement: Additional file 2: — Protocol S1. Scripts for a classification algorithm used to identify beta-barrel core or pore residues. (PDF 29 kb) [file 12858_2015_47_MOESM2_ESM.pdf]

Supporting Information: Protocol S1

Protocol_S1.zip is hosted on **labarchives.com**

URL: https://mynotebook.labarchives.com/share_attachment/Deepesh-notebook/MjMuNHw5MTczMS8xOC0xNC9UcmVlTm9kZS8zNDM2MDM2MTg2fDU5LjQ=

DOI: 10.6070/H4GM8594
