# Supplementary material for: Design of symmetric TIM barrel proteins from first principles
Source: BMC Biochem. 2015 Aug 12;16:18. doi: 10.1186/s12858-015-0047-4 (PMC4531894; doi:10.1186/s12858-015-0047-4)
Supplement: Additional file 3: Figure S1. — Intrastrand and interstrand distances have been described for two parallel beta strands. (PDF 67 kb) [file 12858_2015_47_MOESM3_ESM.pdf]

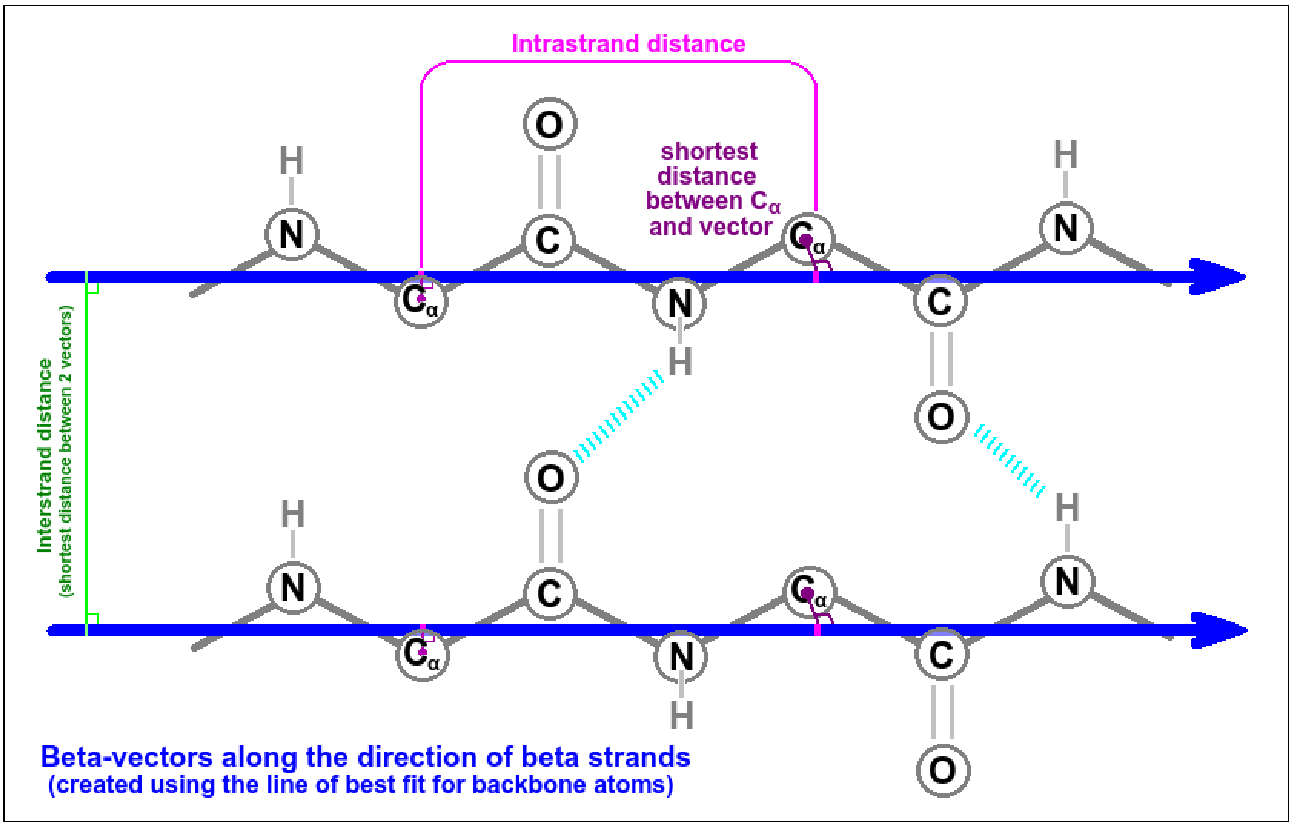

Figure S1. Intrastrand and interstrand distances have been described for two parallel beta strands.
