# Supplementary material for: Design of symmetric TIM barrel proteins from first principles
Source: BMC Biochem. 2015 Aug 12;16:18. doi: 10.1186/s12858-015-0047-4 (PMC4531894; doi:10.1186/s12858-015-0047-4)
Supplement: Additional file 5: — Protocol S2. Scripts for the positive design workflow required to design 4-fold symmetric TIM barrel proteins. (PDF 29 kb) [file 12858_2015_47_MOESM5_ESM.pdf]

Supporting Information: Protocol S2

Protocol_S2.zip is hosted on **labarchives.com**

URL: https://mynotebook.labarchives.com/share_attachment/Deepesh-notebook/MjMuNHw5MTczMS8xOC0xNS9UcmVlTm9kZS8zMjIyNzM1NjM5fDU5LjQ=

DOI: 10.6070/H4BV7DMV
