# Supplementary material for: Design of symmetric TIM barrel proteins from first principles
Source: BMC Biochem. 2015 Aug 12;16:18. doi: 10.1186/s12858-015-0047-4 (PMC4531894; doi:10.1186/s12858-015-0047-4)
Supplement: Additional file 6: — Dataset S1. Structures for 27 (αβ)3 modules of KLGP decarboxylase structural homologues. These modules were used in ab initio folding simulations. (PDF 29 kb) [file 12858_2015_47_MOESM6_ESM.pdf]

Supporting Information: Dataset S1

Dataset_S1.zip is hosted on **labarchives.com**

URL: https://mynotebook.labarchives.com/share_attachment/Deepesh-notebook/MjMuNHw5MTczMS8xOC01L1RyZWVOb2RlLzM1MTYyMTY0OTZ8NTkuNA==

DOI: 10.6070/H4NC5Z69
