# Supplementary material for: Design of symmetric TIM barrel proteins from first principles
Source: BMC Biochem. 2015 Aug 12;16:18. doi: 10.1186/s12858-015-0047-4 (PMC4531894; doi:10.1186/s12858-015-0047-4)
Supplement: Additional file 7: — Text S3. Modeling of exposed hydrophobic patches using Rosetta. (PDF 320 kb) [file 12858_2015_47_MOESM7_ESM.pdf]

Text S3. Modeling of exposed hydrophobic patches using Rosetta.

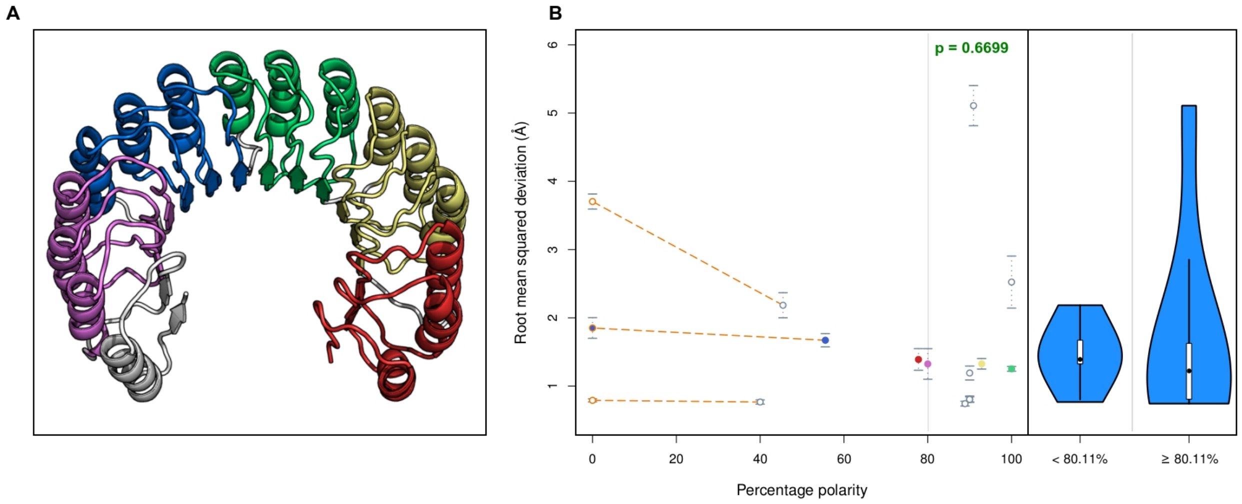

Figure(Text S3). Ab initio modeling of proteins adopting the horseshoe fold. (A) The structure of porcine ribonuclease inhibitor (PDB ID: 2bnh, chain A). The (αβ)3 modules used for ab initio modeling are coloured. (B) Ab initio modeling of select proteins adopting the horseshoe fold. Right: Scatterplots describe the ab initio models mean RMSD distribution (with error bars). Coloured whole dots represent ab initio modeling simulations performed on porcine ribonuclease inhibitor (PDB ID: 2bnh, chain A) (αβ)3 modules. Hollow, grey dots represent simulations performed on other proteins adopting the horseshoe fold. Hollow, orange dots represent simulations performed of isosteric mutants. Dashed, orange lines connect isosteric mutants to their parent modules. A grey line divides the data into relatively apolar pores (<80.11% polarity) and relatively polar pores (≥80.11% polarity). The P value (Welch two sample t-test) is depicted in green. Left: Violin plots depict the data distribution across apolar and polar pores.

The Rosetta energy function strongly emphasizes hydrophobic collapse. It is possible that the energy function is biased against exposed hydrophobic patches that naturally form during the process of protein folding. In order to eliminate this possibility, we performed Rosetta ab initio modeling on $(\alpha\beta)_n$ proteins with naturally exposed hydrophobic patches. We chose the horseshoe fold, which contains solvent-exposed pore beta sheets arranged as a crescent. The buried αβ interface is characteristically leucine-rich. Exposed residues on pore beta sheets vary in hydrophilicity. It is not uncommon to observe hydrophobic patches interspersed among the usually hydrophilic surface. The sequences of 5 $(\alpha\beta)_3$ modules of varying pore polarity (55.56% to 100%) were extracted from porcine ribonuclease inhibitor (PDB ID: 2bnh, chain A), a rather large protein adopting the horseshoe fold (Figure (Text S3)A). These sequences were used for Rosetta ab initio modeling and mean Cα RMSD calculation as previously described for KLGP decarboxylase structural homologues. We observed that all 5 sequences were well-modeled, with a mean RMSD not exceeding 1.67Å. Additional ab initio modeling on $(\alpha\beta)_3$ modules extracted from other proteins adopting the horseshoe fold (PDB ID: 1ogq, chain A; 2ast, chain B; 2omz, chain A) revealed no decrease in foldability with increasing pore polarity. Considering all $(\alpha\beta)_3$ modules, most modules appeared well folded (>90% possessing a mean RMSD of <3Å), and no statistically significant

difference was observed in the foldability between relatively hydrophilic and hydrophobic modules ($p=0.6699$) (Figure (Text S3)B).

We further tested the ability of Rosetta to correctly fold exposed hydrophobic patches by performing tests on 3 naturally hydrophobic $(\alpha\beta)_3$ modules (PDB ID: 2bnh, chain A, module 4, 1ogq, chain A, module1, 2omz, chain A, module 1). Isosteric mutations were performed on the polar pore residues of these 3 modules to decrease their percentage pore polarities to 0%. Ab initio modeling was performed on the resulting sequences. In 2 of 3 cases, Rosetta was able to successfully model $(\alpha\beta)_3$ modules (Figure (Text S3)B), despite the complete absence of polar pore residues. These results indicate that Rosetta ab initio simulations are not biased against exposed hydrophobic patches. Therefore, if $(\alpha\beta)_3$ modules derived from KLGP decarboxylase structural homologues with hydrophobic pores are stable in solution, then there is reason to believe that Rosetta is capable of accurately modeling their structures. Structures for all horseshoe modules described are provided in the supporting information (Dataset S2).
