# Supplementary material for: Design of symmetric TIM barrel proteins from first principles
Source: BMC Biochem. 2015 Aug 12;16:18. doi: 10.1186/s12858-015-0047-4 (PMC4531894; doi:10.1186/s12858-015-0047-4)
Supplement: Additional file 9: Figure S2. — Ab initio modeling of Octarellin V, Octarellin VI, and Symmetrin-1/3 using QUARK. All sequences were divided into four quadrants each before ab initio modeling. For each quadrant, the QUARK ab initio modeling tool generated 10 structures (provided within this dataset). Of the 10 models generated by QUARK, the model most closely resembling the designed topology is displayed. In cases where no topological resemblance was found, the first model is displayed. (A-D) Representative models for all fragments of Octarellin V, arranged from N to C-terminal. The expected alpha/beta secondary structure topology is not observed. Instead, most fragments adopt mostly helical secondary structures. For the fourth quadrant (D), although the correct topology is observed, beta sheets are modeled as disordered loops. (E-H) Representative models for all fragments of Octarellin VI, arranged from N to C-terminal. The second and third quadrants (F-G) display the correct topology. However, beta sheets are poorly modeled. Loops are overextended, cannibalizing residues designed as beta sheets. (I-J) Representative models for the 56-residue quadrants of Symmetrin-1 and Symmetrin-3. In both cases, the correct topology is observed. The ab initio models agree well with the designed structures (depicted in white), and a Kabsch superposition revealed low RMSD values (1.8 Å for Symmetrin-1 and 1.7 Å for Symmetrin-3). Note that designed secondary structure is depicted in colors. Designed alpha helices are colored teal. Designed beta sheets are colored orange. Designed loops are olored green. (PDF 1472 kb) [file 12858_2015_47_MOESM9_ESM.pdf]

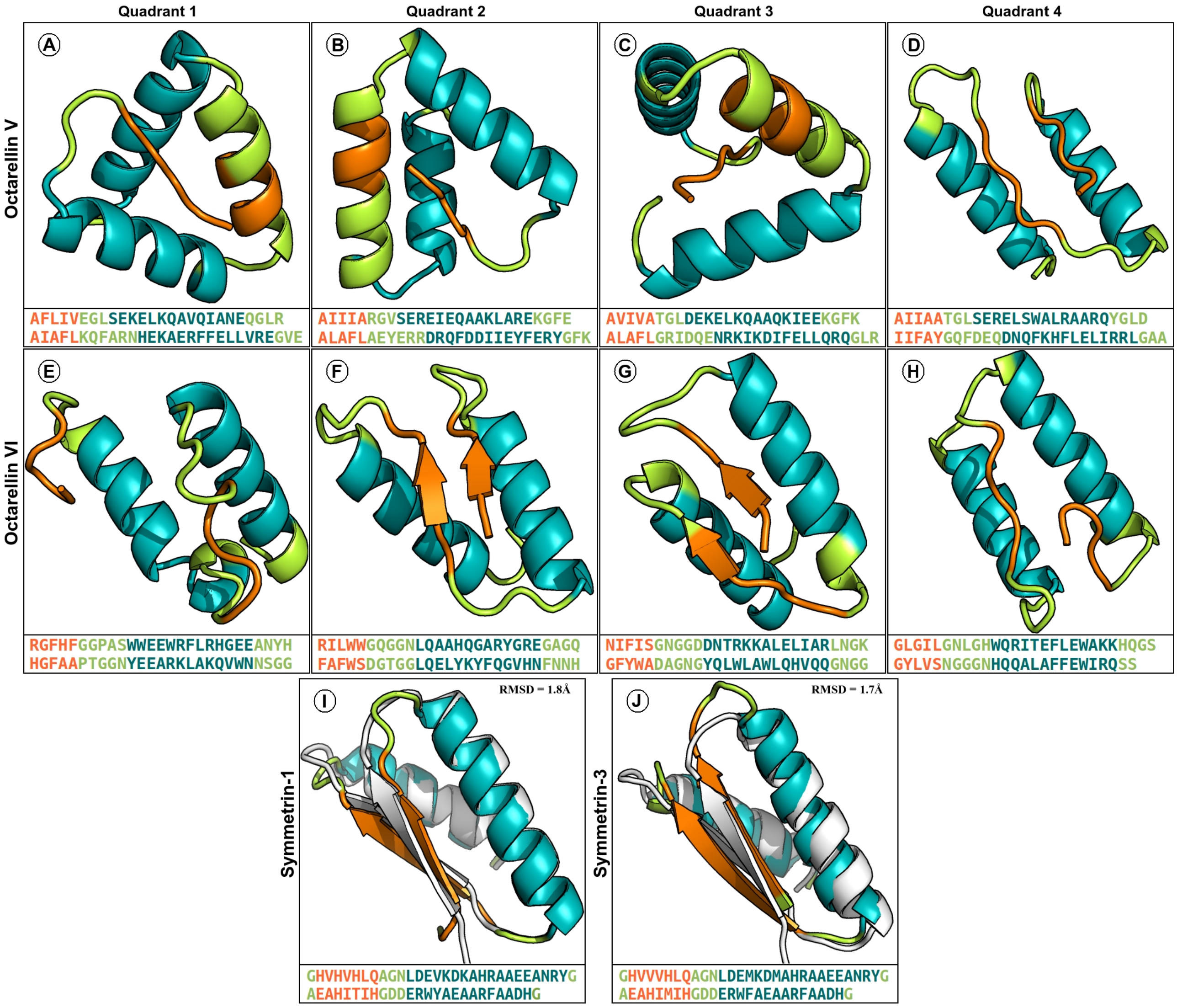

Figure S2. Ab initio modeling of Octarellin V, Octarellin VI, and Symmetrin-1/3 using QUARK. All sequences were divided into four quadrants each before ab initio modeling. For each quadrant, the QUARK ab initio modeling tool generated 10 structures (provided within this dataset). Of the 10 models generated by QUARK, the model most closely resembling the designed topology is displayed. In cases where no topological resemblance was found, the first model is displayed.

(A-D) Representative models for all fragments of Octarellin V, arranged from N to C-terminal. The expected alpha/beta secondary structure topology is not observed. Instead, most fragments adopt mostly helical secondary structures. For the fourth quadrant (D), although the correct topology is observed, beta sheets are modeled as disordered loops.

(E-H) Representative models for all fragments of Octarellin VI, arranged from N to C-terminal. The second and third quadrants (F-G) display the correct topology. However, beta sheets are poorly modeled. Loops are overextended, cannibalizing residues designed as beta sheets.

(I-J) Representative models for the 56-residue quadrants of Symmetrin-1 and Symmetrin-3. In both cases, the correct topology is observed. The ab initio models agree well with the designed structures (depicted in white), and a Kabsch superposition revealed low RMSD values (1.8Å for Symmetrin-1 and 1.7Å for Symmetrin-3).
