# Supplementary material for: Design of symmetric TIM barrel proteins from first principles
Source: BMC Biochem. 2015 Aug 12;16:18. doi: 10.1186/s12858-015-0047-4 (PMC4531894; doi:10.1186/s12858-015-0047-4)
Supplement: Additional file 10: — Dataset S3. QUARK ab initio models for Octarellin V, Octarellin VI, Symmetrin-1, and Symmetrin-3. (PDF 29 kb) [file 12858_2015_47_MOESM10_ESM.pdf]

Supporting Information: Dataset S3

Dataset_S3.zip is hosted on **labarchives.com**

URL: https://mynotebook.labarchives.com/share_attachment/Deepesh-notebook/MjMuNHw5MTczMS8xOC03L1RyZWVOb2RlLzM3MDU0Mjk0NzR8NTkuNA==

DOI: 10.6070/H4CV4FQS
