# Supplementary material for: Design of symmetric TIM barrel proteins from first principles
Source: BMC Biochem. 2015 Aug 12;16:18. doi: 10.1186/s12858-015-0047-4 (PMC4531894; doi:10.1186/s12858-015-0047-4)
Supplement: Additional file 11: Figure S3. — (αβ)2 units are displayed for Symmetrin-1 to Symmetrin-4. We observed that protein stability decreases with decreasing pore polarity. Symmetrin-1 and Symmetrin-2 contain a percentage pore polarities of 100 % and 96.88 % respectively, and are the most stable designs. Symmetrin-3 contains a pore polarity of 75 %, and is relatively unstable, oligomerizing in solution. Symmetrin-4 contains the lowest pore polarity (62.5 %), and is completely insoluble. Hydrophobic residues are coloured yellow. Hydrophilic residues are coloured light teal. (PDF 73 kb) [file 12858_2015_47_MOESM11_ESM.pdf]

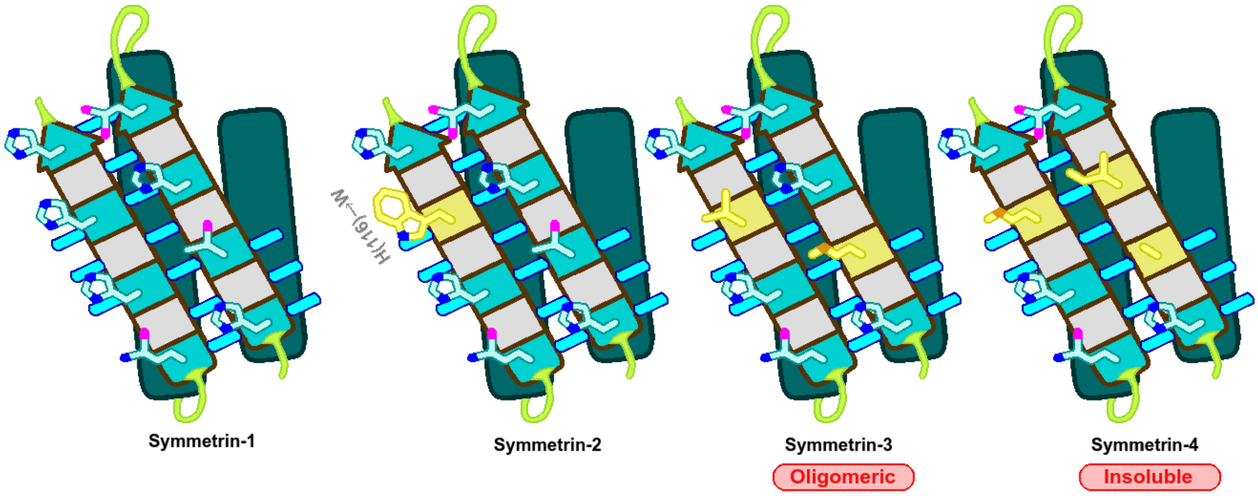

Figure S3. (αβ)$_2$ units are displayed for Symmetrin-1 to Symmetrin-4. We observed that protein stability decreases with decreasing pore polarity. **Symmetrin-1** and **Symmetrin-2** contain a percentage pore polarities of 100% and 96.88% respectively, and are the most stable designs. **Symmetrin-3** contains a pore polarity of 75%, and is relatively unstable, oligomerizing in solution. **Symmetrin-4** contains the lowest pore polarity (62.5%), and is completely insoluble. Hydrophobic residues are coloured yellow. Hydrophilic residues are coloured light teal.
