# Supplementary material for: Design of symmetric TIM barrel proteins from first principles
Source: BMC Biochem. 2015 Aug 12;16:18. doi: 10.1186/s12858-015-0047-4 (PMC4531894; doi:10.1186/s12858-015-0047-4)
Supplement: Additional file 13: Figure S4. — Standard curve for the estimation of the molecular weight of Symmetrin-1 and Symmetrin-3. Proteins similar in molecular weight to Symmetrin-1 and Symmetrin-3 were considered while plotting the standard curve. Albumin (66 kDa), Carbonic Anhydrase (29 kDa), and Cytochrome C (12.4 kDa) were considered while creating the standard curve. (PDF 69 kb) [file 12858_2015_47_MOESM13_ESM.pdf]

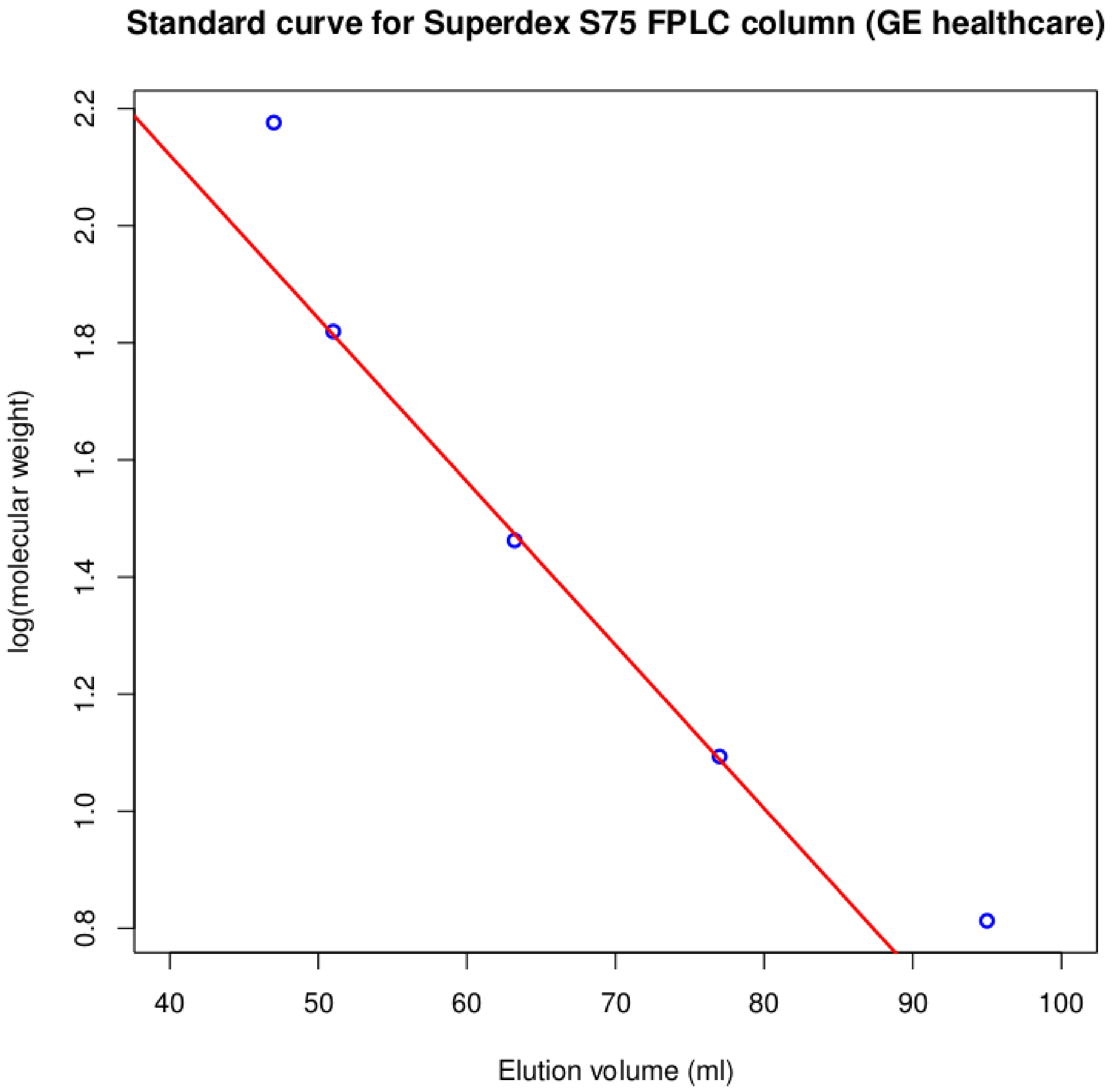

Figure S4. Standard curve for the estimation of the molecular weight of Symmetrin-1 and Symmetrin-3. Proteins similar in molecular weight to Symmetrin-1 and Symmetrin-3 were considered while plotting the standard curve. Albumin (66kDa), Carbonic Anhydrase (29kDa), and Cytochrome C (12.4kDa) were considered while creating the standard curve.
