# Supplementary material for: Design of symmetric TIM barrel proteins from first principles
Source: BMC Biochem. 2015 Aug 12;16:18. doi: 10.1186/s12858-015-0047-4 (PMC4531894; doi:10.1186/s12858-015-0047-4)
Supplement: Additional file 14: Figure S5. — Size exclusion chromatography performed on isolated monomeric Symmetrin-3. Previously isolated monomeric Symmetrin-3 was stored at 4 °C/48 h prior to this experiment. The monomeric Symmetrin-3 species appears to slowly convert to oligomeric forms. (PDF 84 kb) [file 12858_2015_47_MOESM14_ESM.pdf]

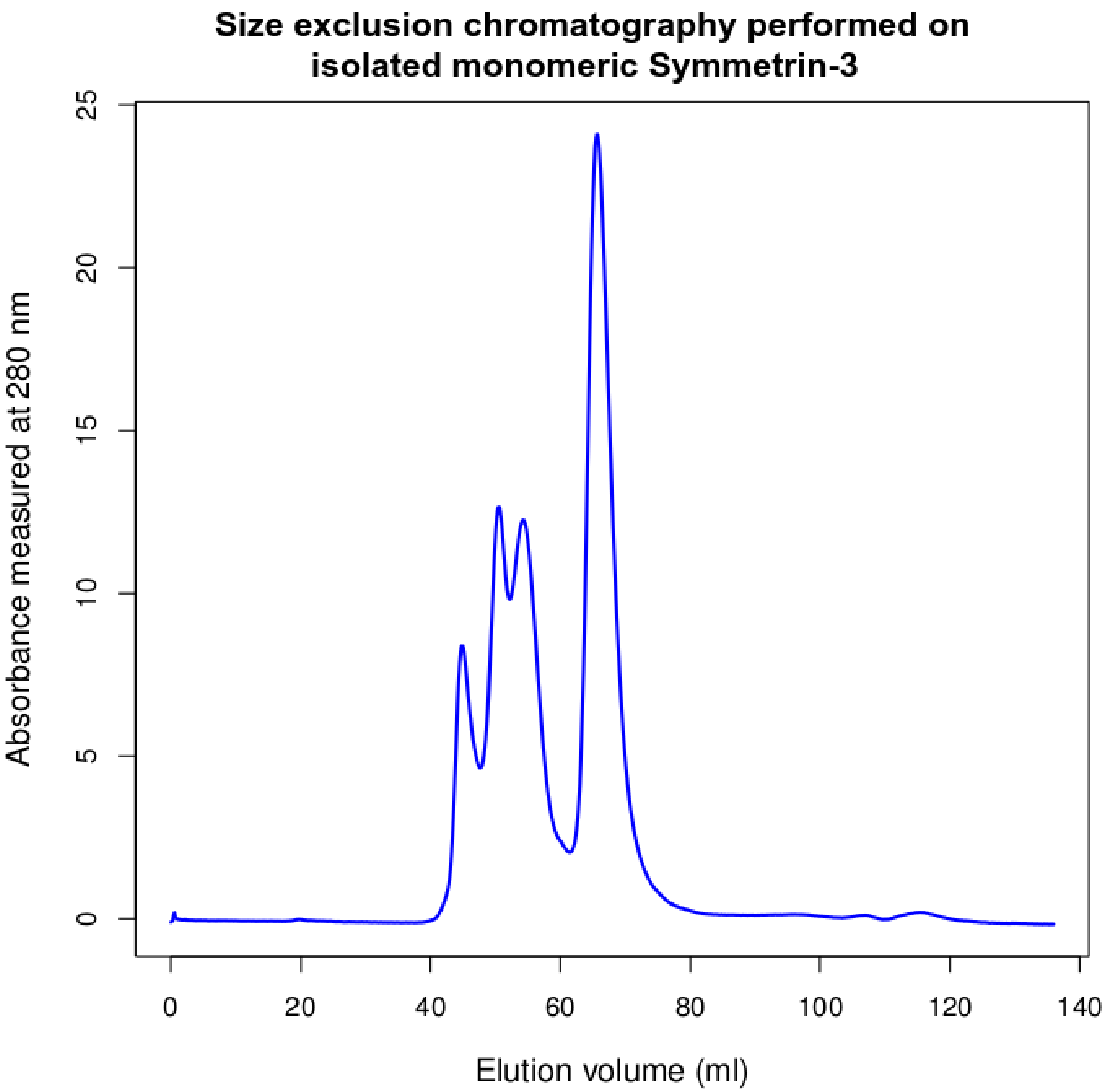

Figure S5. Size exclusion chromatography performed on isolated monomeric Symmetrin-3. Previously isolated monomeric Symmetrin-3 was stored at 4°C/48 hours prior to this experiment. The monomeric Symmetrin-3 species appears to slowly convert to oligomeric forms.
